# Sequential salvage systemic therapy after immunotherapy in head and neck cancer: a real-world study

**DOI:** 10.3389/fonc.2025.1719793

**Published:** 2026-01-09

**Authors:** Santiago Cabezas-Camarero, Salomé Merino-Menéndez, María Nieves Cabrera-Martín, Pablo Pérez-Alonso, Miguel J. Sotelo, Pedro Pérez-Segura

**Affiliations:** 1 Department of Medical Oncology, Instituto de Investigación Sanitaria San Carlos (IdISSC), Hospital Clínico Universitario San Carlos, Madrid, Spain; 2 Department of Medical Oncology, IOB Institute of Oncology – Madrid, Madrid, Spain; 3 Department of Radiology, Instituto de Investigación Sanitaria San Carlos (IdISSC), Hospital Clínico Universitario San Carlos, Madrid, Spain; 4 Department of Nuclear Medicine, Instituto de Investigación Sanitaria San Carlos (IdISSC), Hospital Clínico Universitario San Carlos, Madrid, Spain; 5 Department of Pathology, Hospital Clínico Universitario San Carlos, Madrid, Spain; 6 Department of Medical Oncology, Aliada Cancer Center, Lima, Peru; 7 Department of Medical Oncology, Clínica San Felipe, Lima, Peru; 8 Department of Medical Oncology, Hospital María Auxiliadora, Lima, Peru

**Keywords:** cetuximab, head and neck cancer, immune checkpoint inhibitors, rechallenge, sequential therapy

## Abstract

**Background:**

In recurrent/metastatic (R/M) squamous cell carcinoma of the head and neck (SCCHN) there is no established standard therapy after progression to immune checkpoint inhibitors (ICI). Retrospective and limited prospective studies have suggested that salvage chemotherapy after ICI (SCAI) may outperform historical pre-ICI data. We evaluated cetuximab-based SCAI outcomes in a real-world setting.

**Methods:**

Objective response rate (ORR), median duration of response (DoR), and median best percentage change in target lesions (PCTL) by Response Evaluation Criteria In Solid Tumors version 1.1 (RECIST 1.1), and median progression-free survival (PFS) and overall survival (OS) with SCAI and with last chemotherapy before immunotherapy (LCBI) were assessed. Adverse events (AEs) by Common Terminology Criteria for Adverse Events (CTCAE) 5.0 were evaluated during SCAI, as well as treatment exposure to cetuximab-based therapy and to ICI across the whole R/M setting.

**Results:**

Among 80 patients, 96% and 100% received cetuximab-based first and second SCAI, respectively, primarily as weekly (90%–94%) regimens, as follows. First SCAI: ORR of 59% (41/70; 31 partial and 10 complete responses), PCTL of −57% (range, −30% to −100%), DoR of 9.4 months, and PFS and OS of 5.9 and 12.4 months, respectively; first-line OS of 24 months. Cetuximab-based exposure (256 days) significantly exceeded ICI exposure (168.5 days; p = 0.038). LCBI-treated (n = 22): ORR of 36% (LCBI) *vs*. 47% (SCAI), PFS 8 months (LCBI) *vs*. 4.4 months (SCAI); first-line OS of 25.9 months. Second SCAI (n = 17): ORR of 30% (3/10), PFS and OS of 3.5 and 7 months, respectively; first-line OS of 21.7 months. Grade 1–2 AEs, 100%; grade 3–5 AEs, 38.7% (first SCAI) and 47% (second SCAI) with no toxic deaths.

**Conclusion:**

Cetuximab-based SCAI post-ICI showed high rates of response, which were durable and profound, and improved survival compared to historical data, with modest efficacy in re-sequenced patients. Time on cetuximab was significantly longer than on ICI. These results should be confirmed in a larger prospective study.

## Introduction

The introduction of immune checkpoint inhibitors (ICIs) in the past decade has improved the survival of patients with recurrent/metastatic (R/M) squamous cell carcinoma of the head and neck (SCCHN), but in most patients, this benefit is modest, with a median overall survival (OS) below 15 months in the platinum-sensitive setting and below 8 months in the first-line platinum-refractory population in phase III randomized trials (1, 2). In these studies, less than 50% of the patients received a second line of therapy after progression to ICI, and among those receiving a second line, there was a high heterogeneity in the regimens administered, mainly consisting of monotherapies with platinum, taxanes, or anti-Epidermal Growth Factor Receptor (EGFR) agents. The absence of a globally standardized second-line treatment for R/M SCCHN stems from a lack of prospective, randomized, evidence-based studies demonstrating significant survival benefits for any regimen (3, 4). Recent retrospective and limited prospective studies have suggested that salvage chemotherapy after ICI (SCAI) may yield higher objective response rates (ORRs) and improved progression-free survival (PFS) and OS compared to historical pre-ICI data (5–9). Notably, cetuximab plus taxane-based SCAI, widely used as a second-line regimen before the immunotherapy era, has shown promise (8–15). The enhanced activity of cetuximab in this setting may be driven by its ability to modulate the tumor microenvironment, promoting immune cell infiltration and synergizing with prior ICI-induced immune activation. More specifically, there is some evidence that the enhanced activity and efficacy of cetuximab-based treatments may rely upon the promotion of antibody-dependent cellular cytotoxicity (ADCC) (16, 17). In our previously published series of 23 patients treated with cetuximab-based SCAI, ORR was 56.5% with a median PFS and OS of 6 and 12 months, respectively, and an OS since first line of 28 months (14). Subsequent studies have further supported the activity and tolerability of taxanes and cetuximab post-ICI (8–15). Nevertheless, key questions remain unanswered due to the predominance of small, retrospective studies. This study aimed to update our previous series by evaluating response and survival outcomes in patients treated with first and second SCAI, as well as assessing the cumulative impact of ICI and cetuximab across the entire treatment journey.

## Materials and methods

### Patients and study design

This retrospective study included patients with R/M SCCHN treated at Hospital Universitario Clínico San Carlos who received SCAI following radiological and clinical progression on PD-1/PD-L1 inhibitors. This study updates a previously published series (14). SCAI was defined as any salvage systemic therapy administered after an objective radiological or clinical progression to an immediately prior line with anti**-**PD(L)1 agents. First and second SCAI occurred the first or second time SCAI was administered, respectively. Last chemotherapy before immunotherapy (LCBI) was defined as those patients treated with a non-ICI line in the R/M setting before the first SCAI. Patients were eligible irrespective of the number of pre-SCAI or post-SCAI lines received. **Supplementary Figure S1** depicts the allocation of patients in each category (LCBI, first SCAI, and second SCAI) during the present study.

Baseline characteristics were analyzed before initiating LCBI, first SCAI, and second SCAI. Outcomes assessed included ORR, disease control rate (DCR), percentage change in target lesions (PCTL) per Response Evaluation Criteria In Solid Tumors version 1.1 (RECIST 1.1), PFS, overall survival (OS), and toxicity per National Cancer Institute Common Terminology Criteria for Adverse Events (CTCAE) v5.0 (18, 19). Outcomes for the LCBI were also evaluated.

### Endpoints

Primary endpoints were ORR, PCTL, and OS during the first SCAI. Secondary endpoints included DCR, PFS, and OS by combined positive score (CPS) status, and safety during the first SCAI; ORR, PFS, and OS for LCBI and the first SCAI in LCBI-pretreated patients; OS in first line; and comparison of treatment exposure to cetuximab-based therapy and ICI during the whole R/M setting. Exploratory endpoints included response, survival, and toxicity during the second SCAI. PFS for SCAI and LCBI was defined as the time from the start of treatment to disease progression or death from any cause. OS for SCAI was defined as the time from SCAI initiation to death from any cause. OS in first-line treatment was defined as the time from the start of first-line therapy to death from any cause. Response dynamics and treatment duration were visualized using Trial Reporting in Immuno-Oncology (TRIO) guideline-compliant plots (20).

### Response assessment

Tumor response was assessed using computed tomography (CT) every 8–12 weeks per local protocol. Two head and neck cancer radiologists independently evaluated responses, with consensus reached through reassessment in cases of disagreement. Response categories were defined per RECIST 1.1 (18). Complete response (CR) was defined as the disappearance of all target lesions (TLs) and non-target lesions (non-TLs). Partial response (PR) required a ≥30% decrease in the sum of TL diameters. Progressive disease (PD) was defined as a ≥20% increase in the sum of TL diameters or the appearance of new metastatic lesions. Stable disease (SD) was defined as a change in TL diameters ranging from a <30% decrease to a <20% increase.

### Biomarkers

PD-L1 expression was assessed by immunohistochemistry (IHC) using the anti-PD-L1 antibody (clone 22C3, 1:50 dilution; Dako North America, Carpinteria, CA, USA) and scored using the CPS. CPS was calculated as [(PD-L1-positive tumor cells + PD-L1-positive mononuclear inflammatory cells)/total tumor cells] × 100, with positive PD-L1 expression defined as any membranous staining in tumor cells or membranous/cytoplasmic staining in stromal mononuclear immune cells (21). Human papillomavirus (HPV) status was evaluated by IHC using a p16 monoclonal antibody (clone JC8, 1:25 dilution; Dako North America, Carpinteria, CA, USA). Positive p16 expression was defined as ≥70% diffuse tumor cell staining (22).

### Statistics

Demographic and clinicopathological data underwent a descriptive evaluation. PFS and OS were calculated via the Kaplan–Meier approach, while comparisons across treatment groups employed the log-rank test. Qualitative variables were compared using the χ^2^ test or Fisher’s exact test, favoring non-parametric methods for data lacking normal distribution. Univariate and multivariate Cox regression analyses were conducted to evaluate the association of demographic, clinical, treatment-related, and activity (response) variables with PFS since first systemic therapy (SCAI), OS since the first SCAI, and OS since first-line treatment in the total population (n = 80).

All statistical computations were carried out in R version 2024.12.0 + 467 (2024.12.0 + 467) through RStudio (Posit Software, PBC; https://www.r-project.org).

### Ethical considerations

The Institutional Review Board at Hospital Clínico Universitario San Carlos approved the study, which adhered to the Declaration of Helsinki guidelines. Informed consent was not required, given the retrospective nature of the analysis, which relied on pre-existing clinical and administrative records. Some patients received anti-PD(L)1 agents pre-SCAI within clinical trials, all terminated and with their databases closed at the time of publication of this manuscript (NCT02499328, NCT02551159, NCT05061420, NCT04634825, and NCT04590963). AstraZeneca gave consent for the use of the efficacy and toxicity data of three patients (Id 78, 79, and 80) during their participation in a terminated and already closed clinical trial of post-ICI cetuximab ± monalizumab (NCT04590963) (23).

## Results

### Baseline characteristics

Between March 2016 and February 2025, 80 patients with R/M SCCHN who received at least the first SCAI were identified. At the first SCAI initiation, the median age was 69 years, 64% were male, and 57.5% had Eastern Cooperative Oncology Group Performance Status (ECOG PS 2) (remainder ECOG PS 1). Primary tumor sites were oral cavity (54%), oropharynx (17.5%), and larynx (15%). Most patients had locally advanced disease at initial diagnosis (77.5%), with 86% receiving prior radiotherapy and systemic therapy and 52% undergoing surgery. At relapse, 13.75% were platinum-refractory. The median CPS was 20 (range, 0–100). SCAI was administered as the second, third, or fourth line in 60%, 32.5%, and 7.5% of patients, respectively.

Of 22 patients receiving LCBI, the median age was 60 years, 59% were male, and ECOG PS was 0 (59%) or 1 (41%). Most had locally advanced disease at diagnosis (87%), with primary tumors in the oral cavity (54.5%), and received surgery (64%), radiotherapy (77.5%), or systemic therapy (82%). At relapse, 18% were platinum-refractory. The median CPS was 20 (range, 0–100). LCBI was administered as first, second, or third line in 18%, 64%, and 18% of patients, respectively.

Thirty-one patients (38.75%) received ICI rechallenge after progression to the first SCAI.

Seventeen patients received a second SCAI after anti-PD-(L)1 rechallenge, having progressed radiologically and symptomatically on prior ICI with interval chemotherapy between ICI treatments. The median age was 63 years, 59% were male, 82% had ECOG PS 2, and 76.5% had oral cavity primary tumors. At relapse, 18% were platinum-refractory, and the median CPS was 20 (range, 0–90). Additional baseline characteristics are summarized in **Table 1**.

**Table 1:** Summary of baseline characteristics.

Variable		LCBI	1st SCAI	1st SCAI (LCBI)	2nd SCAI
N		22	80	22	17
Age (1st SCAI)		60 (36–97)	69 (36–97)	60 (36–93)	63 (50–91)
ECOG PS	0	13 (59%)	0/80 (0%)	0	0
	1	9 (41%)	34 (42.5%)	10 (45.5%)	3 (18%)
	2	0	46 (57.5%)	12 (54.5%)	14 (82%)
Smoking		14 (64%)	56 (70%)	14 (64%)	10 (59%)
Alcohol		10 (45.5%)	41 (51%)	10 (45.5%)	9 (53%)
Sex (1st SCAI)	Male	13 (59%)	51 (63.75%)	13 (59%)	10 (59%)
	Female	9 (41%)	29 (36.25%)	9 (41%)	7 (42%)
Anatomic subsite	Oral cavity	12 (54.5%)	43 (54%)	12 (54.5%)	13 (76.5%)
	Oropharynx HPV−	1 (4.5%)	9 (11.25%)	1 (4.5%)	1 (6%)
	Oropharynx HPV+	0	5 (6.25%)	0	0
	Larynx	5 (23%)	12 (15%)	5 (23%)	2 (12%)
	Hypopharynx	2 (9%)	6 (7.5%)	2 (9%)	1 (6%)
	Unknown primary	2 (9%)	5 (6.25%)	2 (9%)	0
	Sinonasal	0	2 (2.5%)	0	0
Stage at initial diagnosis (AJCC 8th Ed.)	I	1 (4.5%)	1 (1.25%)	1 (4.5%)	0
	II	2 (9%)	8 (10%)	2 (9%)	3 (18%)
	III	3 (14%)	9 (11.25%)	3 (14%)	1 (6%)
	IVA	13 (59%)	37 (46.25%)	13 (59%)	5 (29%)
	IVB	3 (14%)	16 (20%)	3 (14%)	5 (29%)
	IVC	0	9 (11.25%)	0	3 (18%)
Treatment at initial diagnosis	Surgery	14 (64%)	40/77 (52%)	14 (64%)	11 (65%)
	RT alone	5 (23%)	10/72 (14%)	5 (23%)	3 (18%)
	RT + Chemotherapy (CTx)	12 (54.5%)	52/72 (72%)	12 (54.5%)	11 (65%)
	Induction CTx	6 (27%)	27/73 (37%)	6 (28%)	5 (29%)
	RT + platinum	5 (23%)	15/72 (21%)	5 (23%)	5 (29%)
	RT + cetuximab	7 (32%)	37/72 (51%)	7 (32%)	6 (35%)
Platinum-refractory at relapse		4 (18%)	11 (13.75%)	4 (18%)	3 (18%)
PD-L1 (CPS)		20 (0–100)	20 (0–100)	20 (0–100)	20 (0–90)
ICI pre-SCAI	Anti-PD1	–	68 (85%)	20 (91%)	11 (65%)
	Anti-PD-L1	–	12 (15%)	2 (9%)	6 (35%)
Cetuximab in R/M setting pre-SCAI		–	20 (25%)	20 (91%)	17 (100%)
Cetuximab-based SCAI or LCBI		20 (91%)	77 (96%)	21 (95%)	17 (100%)
SCAI or LCBI line	1st	4 (18%)	–	–	–
	2nd	14 (64%)	48 (60%)	–	–
	3rd	4 (18%)	26 (32.5%)	4 (18%)	–
	4th	0	6 (7.5%)	14 (64%)	13 (76.5%)
	5th	0	0	4 (18%)	4 (23.5%)
SCAI or LCBI modality	Weekly	12 (54.5%)	72 (90%)	18 (82%)	16/17 (94%)
	Three-weekly	10 (45.5%)	8 (10%)	4 (18%)	1/17 (6%)
SCAI or LCBI type	Erbitax	10 (45.5%)	51 (63.75%)	13 (59%)	5 (29%)
	Carbitax	7 (32%)	10 (12.5%)	1 (4.5%)	3 (18%)
	Cetuximab + wkCDDP	2 (9%)	3 (3.75%)	3 (13.5%)	8 (47%)
	Cetuximab + wkCarbo	0	1 (1.25%)	1 (4.5%)	0
	Cetuximab + Anti-PD1	0	2 (2.5%)	0	0
	Extreme	0	3 (3.75%)	3 (14%)	1 (6%)
	TPEX	1 (4.5%)	2 (2.5%)	0	0
	Cetuximab monotherapy	0	5 (6.25%)	0	0
	3wkCDDP–docetaxel	0	2 (2.5%)	0	0
	3wkCarbo–paclitaxel	1 (4.5%)	1 (1.25%)	1 (4.5%)	0
	TPF	1 (4.5%)	–	0	0
	Anti-PD-L1 (CT)	–	–	–	–
	Anti-PD-L1 + Other ICI (CT)	–	–	–	–
	Anti-PD1 + CTx	–	0	0	0
Post-SCAI/LCBI lines	Median no.	2 (2–5)	0 (0–5)	0 (0–3)	1 (0–3)
	0	–	46 (57.5%)	12 (54.5%)	8 (47%)
	1	–	15 (18.75%)	6 (27%)	6 (35%)
	2	12	9 (11.25%)	3 (14%)	2 (12%)
	3	6	7 (8.75%)	1 (4.5%)	1 (6%)
	4	3	2 (2.5%)	0	0
	5	1	1 (1.25%)	0	0

AJCC, American Joint Committee on Cancer; Carbitax, cetuximab plus weekly paclitaxel and weekly carboplatin; CPS, combined positive score; CTx, chemotherapy; (CT), clinical trial; ECOG PS, Eastern Cooperative Oncology Group Performance Status; Erbitax, cetuximab + weekly paclitaxel, cetuximab + platinum + 5-fluorouracil; HPV, human papillomavirus; ICI, immune checkpoint inhibitor; LCBI, last chemotherapy before immunotherapy; PD1, programmed-death receptor 1; PD-L1, programmed-death receptor 1 ligand; RT, radiotherapy; SCAI, salvage chemotherapy after immunotherapy; TPEx, cetuximab + platinum + docetaxel; TPF, platinum + 5-fluorouracil + docetaxel; wkCarbo, weekly carboplatin; wkCDDP, weekly cisplatin; 3wkCarbo, three-weekly carboplatin; 3wkCDDP, three-weekly cisplatin; (-), not applicable; R/M, recurrent/metastatic.

### Regimens used

#### Anti-PD(L)1 inhibitors

Prior to first SCAI, 85% of patients received anti-PD-1 agents, with anti-PD-L1 inhibitors used in the remainder. Before second SCAI, 65% received anti-PD-1 agents, and anti-PD-L1 inhibitors in the remainder (**Table 1**).

#### Last chemotherapy before immunotherapy

The most common LCBI regimen was Erbitax (weekly cetuximab: 400 mg/m^2^ loading dose, 250 mg/m^2^ maintenance; plus weekly paclitaxel 80 mg/m^2^) for 8–12 weeks, followed by biweekly cetuximab (500 mg/m^2^) maintenance (**Table 1**).

#### First salvage chemotherapy after immunotherapy

Weekly regimens were used in 90% of the first SCAI cases, with Erbitax (64%) as the most common, followed by Carbitax (Erbitax plus weekly carboplatin AUC 2) in 12.5% (**Table 1**).

#### Second salvage chemotherapy after immunotherapy

All but one patient received weekly regimens for the second SCAI. The most common was cetuximab plus weekly cisplatin (40 mg/m^2^) (47%) for 8–12 weeks, followed by biweekly cetuximab (500 mg/m^2^). The second most used regimen was Erbitax (29%) (**Table 1**).

### Activity and efficacy during first SCAI and LCBI

#### Response during first SCAI and LCBI

**Figure 1:**
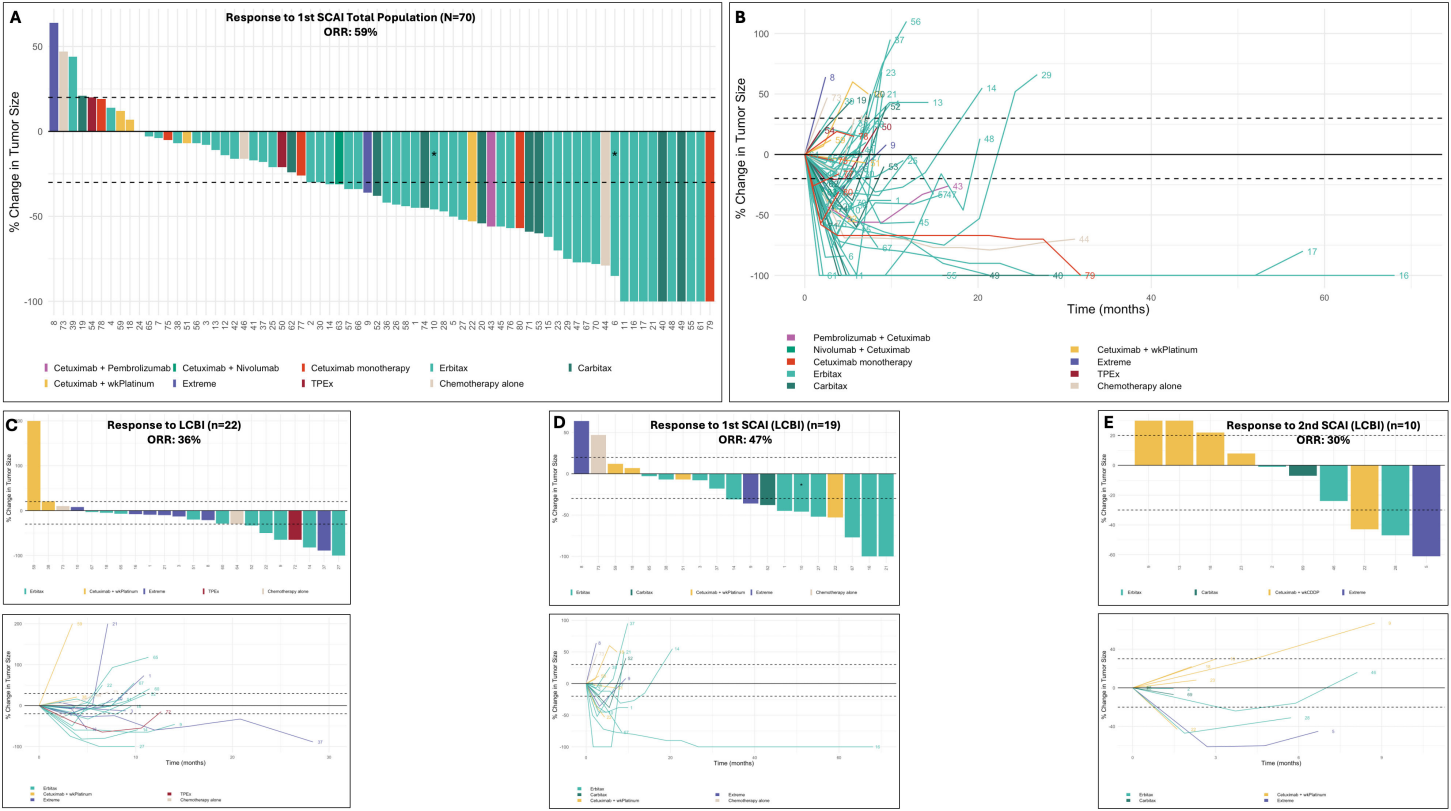
Waterfall and spider plots depicting the response to first SCAI **(A, B)**, LCBI **(C)**, first SCAI in LCBI pre-treated patients **(D)**, and second SCAI **(E)**. Asterisks (*) indicate patients who achieved an overall progressive disease, despite responding in target lesions, due to the appearance of new non-measurable (bone) metastatic lesions. Patient 53 received a single first SCAI cycle of three-weekly carboplatin–paclitaxel, but due to clinical overt progressive disease, was switched to Carbitax, showing a rapid clinical response and radiological 60% tumor reduction at the first reevaluation after starting Carbitax. Patients 78 and 79 received cetuximab + monalizumab, and patient 80 received cetuximab + placebo, all three within a clinical trial (NCT04590963) and coded in panels A and B within the group of cetuximab monotherapy due to the futility of the experimental drug within the trial (publication of the data was allowed by the trial sponsor, AstraZeneca). For more details, please refer to the main text. Carbitax, cetuximab + weekly paclitaxel + weekly carboplatin; (CT), clinical trial; CTx, chemotherapy; Erbitax, cetuximab + weekly paclitaxel; Extreme, cetuximab + platinum + 5-FU; ICI, immune checkpoint inhibitor; TPEx, cetuximab + docetaxel + platinum; wkPlatinum, weekly platinum; LCBI, last chemotherapy before immunotherapy; SCAI, salvage chemotherapy after ICI; 5-FU, 5-fluorouracil.

In the total population, first SCAI yielded an objective response rate (ORR) of 59% and 61% in TLs among 70 evaluable patients (**Figure 1**, **Supplementary Figure S1**). Ten patients were not evaluable because either the pre-SCAI or post-SCAI scan was not performed. PCTL among objective responders was −57% (range, −30% to −100%). The median duration of response (DoR) among objective responders in TL (n = 43) was 9.4 months (95%CI 7.53–20.15). Among 22 evaluable LCBI-pretreated patients, LCBI achieved an ORR of 36% (overall and TLs), while first SCAI in 19 evaluable LCBI-pretreated patients resulted in ORRs of 47% (overall) and 53% (TLs) (**Table 2**, **Figure 1**, **Supplementary Figures S2 and S5**).

**Table 2:** Summary of objective responses and survival analyses.

Objective response			
Setting	Response	Target lesions	Overall response*
LCBI (1st SCAI)	CR	1	1
	PR	7	7
	SD	12	11
	PD	2	3
	ORR	8/22 (36%)	8/22 (36%)
1st SCAI (Total pop)—BOR	CR	11	9
	PR	32	32
	SD	20	20
	PD	7	9
	ORR	43/70 (61%)	41/70 (59%)
	DoR	9.4 m (7.53–20.15)	–
1st SCAI (Erbitax pop)—BOR	CR	7	7
	PR	22	20
	SD	13	13
	PD	1	3
	ORR	29/43 (67%)	27/43 (63%)
	DoR	9.01 m (7.1–57.5)	–
1st SCAI (LCBI)—BOR	CR	2	2
	PR	8	7
	SD	6	6
	PD	3	4
	ORR	10/19 (53%)	9/19 (47%)
2nd SCAI—BOR	CR	0	0
	PR	3	3
	SD	4	3
	PD	3	4
	ORR	3/10 (30%)	3/10 (30%)

Survival			
Setting	Population	OS	
1st line	Follow-up	22.9 m (0.8–102)	
	Survival	24 m (20.9–30)	
	Survival (CPS ≥20 *vs*. <20)	29.3 *vs* 23.9 m p-Value: 0.97	
	Survival (2nd *vs*. 3rd/4th line)	16.6 *vs*. 26.2 m p-Value: 0.16	
	Follow-up (Erbitax pop.)	24.7 m (0.9–110)	
	Survival (Erbitax pop.)	24 m (19.4–30)	
	Survival (CPS ≥20 *vs*. <20) (Erbitax pop.)	29.3 *vs* 23.9 m p-Value: 0.902	
	Follow-up (LCBI pop.)	26.1 m (0.8–102)	
	Survival (LCBI pop.)	25.9 m (23.9–40)	
	Survival (2nd SCAI)	21.7 m (17.9–31)	

Setting	Population	PFS	OS
LCBI	Follow-up	26.2 m (0.8–102)	
	Survival	8 (5.5–10.5)	25.9 m (23.9–40)
1st SCAI	Follow-up	10.1 m (0.1-71.6)	
	Survival	5.92 m (4.3–8.2)	12.4 m (9–14)
	Survival (CPS ≥20 *vs*. <20)	6.7 *vs*. 4.8 m p-Value: 0.38	14.1 *vs*. 9.4 m p-Value: 0.62
	Follow-up (Erbitax pop.)	12.4 m (0.7–100)	
	Survival (Erbitax pop.)	6.05 m (4.5–9.4)	12.4 m (9.4–17.4)
	Survival (CPS ≥20 *vs*. <20) (Erbitax pop.)	14 *vs*. 5.92 m p-Value: 0.168	15.45 *vs*. 9.4 m p-Value: 0.645
	Follow-up (LCBI pop.)	9.5 m (0.1-71.6)	
	Survival (LCBI pop.)	4.4 m (2.5–8.7)	9.4 m (6.8–15.4)
2nd SCAI	Follow-up	5.5 m (0.6–83.2)	
	Survival	3.5 m (1.2–7.1)	7.1 m (7.1–NR)

BOR, best objective response; CR, complete response; LCBI, last chemotherapy before immunotherapy; m, months; ORR, objective response rate; OS, overall survival; PD, progressive disease; PFS, progression-free survival; pop, population; PR, partial response; SCAI, salvage chemotherapy after immunotherapy; SD, stable disease; RECIST 1.1, Response Evaluation Criteria In Solid Tumors version 1.1.

*Overall response refers to RECIST 1.1 objective response in target and non-target lesions. Follow-up times are presented as median (Min–Max). Survival times are presented as median (95%CI).

#### Response with Erbitax during first SCAI

Among 43 evaluable patients treated with Erbitax as first SCAI, overall ORR was 63%, and TL ORR was 67%. PCTL among objective responders in TL was −57% (range, −30% to −100%). The median DoR among objective responders in TL (n = 28) was 9.01 months (95%CI 7.1–57.5). ORR with Erbitax as 2nd-line (n = 27) and ≥3rd-line (n = 16) were 59% and 69%, respectively. In patients pretreated with ICI monotherapy, ORR was 61% for both groups. Among seven patients pretreated with anti-PD-1 plus chemotherapy before SCAI, ORR with Erbitax was 71% (**Table 2**, **Supplementary Figures S3–5**).

#### Survival during first SCAI and LCBI

**Figure 2:**
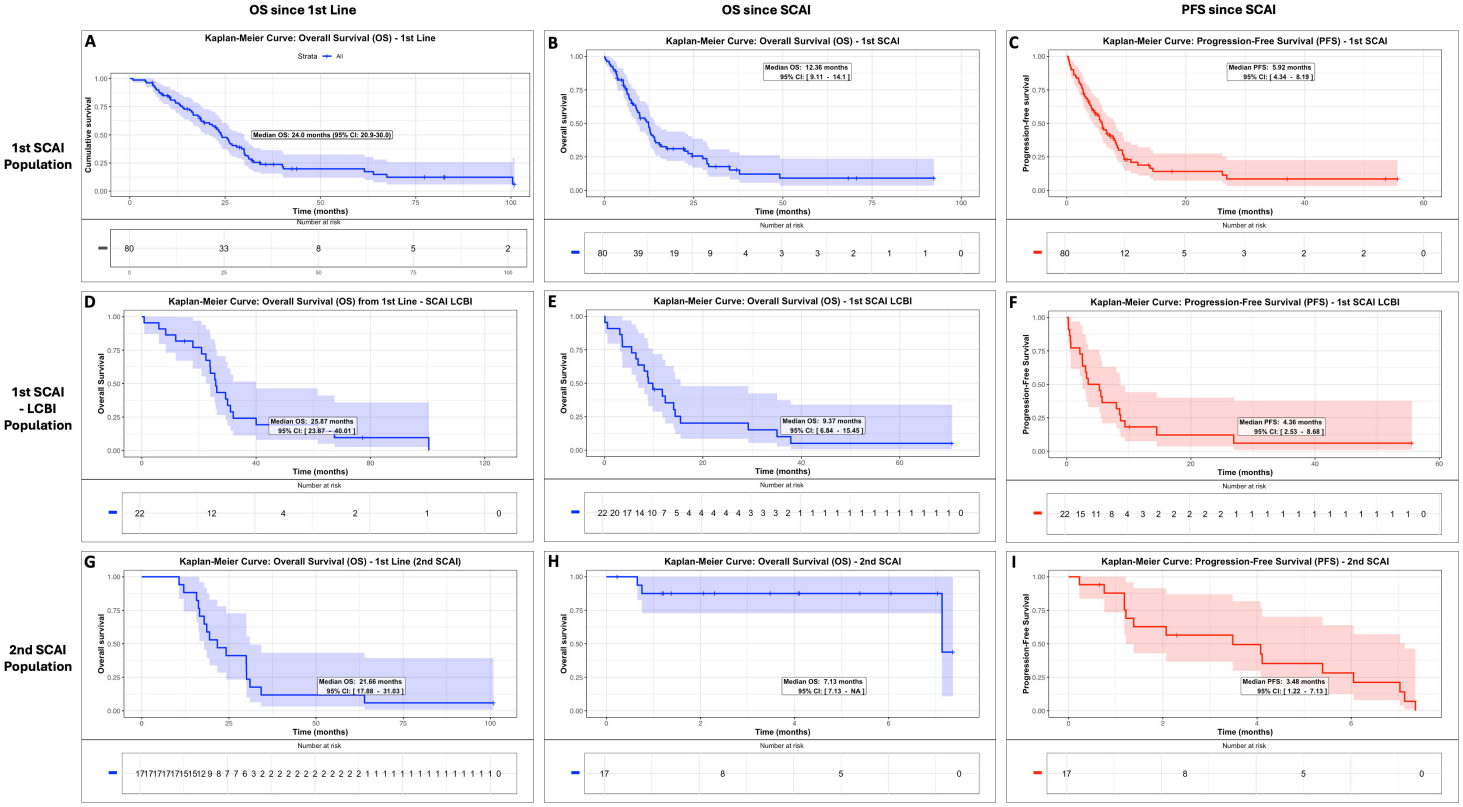
Survival of patients since first line, first SCAI, and second SCAI. Overall survival (OS) and progression-free survival (PFS) in the first SCAI population **(A–C)**, in the first SCAI–LCBI-treated population **(D–F)**, and in the second SCAI population **(G–I)**. LCBI, last chemotherapy before immunotherapy; SCAI, salvage chemotherapy after immunotherapy.

In the total population, median PFS and OS for the first SCAI were 5.9 and 12.4 months, respectively, with no differences by PD-L1 CPS status (PFS, p = 0.38; OS, p = 0.62). For LCBI, median PFS and OS were 8 and 25.9 months, respectively. In LCBI patients, first SCAI yielded median PFS and OS of 4.4 and 9.4 months, respectively (**Table 2**, **Figure 2**, and **Supplementary Figure S6**).

#### Survival with Erbitax during first SCAI

For Erbitax-treated patients, median PFS and OS during first SCAI were 6.05 and 12.4 months, respectively, with no differences by PD-L1 CPS status (PFS, p = 0.168; OS, p = 0.645) (**Table 2**, **Supplementary Figure S7**).

#### Case examples during first SCAI

The case examples of two patients treated with Erbitax (Id 16 and Id 17) and one patient treated with cetuximab plus monalizumab (Id 79) as first SCAI can be found in **Supplementary Figures S8–S10**.

### Activity and efficacy during second SCAI

#### Response during second SCAI

Among 10 evaluable patients, ORR (overall and in TL) during second SCAI was 30% (**Figure 1**, **Supplementary Figure S2**).

#### Survival during second SCAI

Median PFS and OS since second SCAI were 3.5 and 7.1 months, respectively (**Table 2**; **Figure 2**).

#### Case example during second SCAI

A case example of one of the patients (Id 5) treated with the first and second SCAI can be found in **Supplementary Figure S11**.

### Overall survival since first line

After a median follow-up of 22.9 months, median OS since first-line therapy in the total population was 24 months. No differences in OS were observed by PD-L1 expression (CPS) status (p = 0.97), or according to first SCAI line (2nd *vs*. >3rd line; p = 0.16). Survival outcomes are summarized in **Table 2**, **Figure 2**, and **Supplementary Figures S6 and S7**.

### Univariate and multivariate survival analyses

When conducting uni- and multivariate analyses, there were no statistically significant associations between demographic and activity (response) variables for PFS and OS during first SCAI or for OS since first line in the total population (**Supplementary Tables S1–S3**).

### Treatment exposure by regimen type

**Figure 3:**
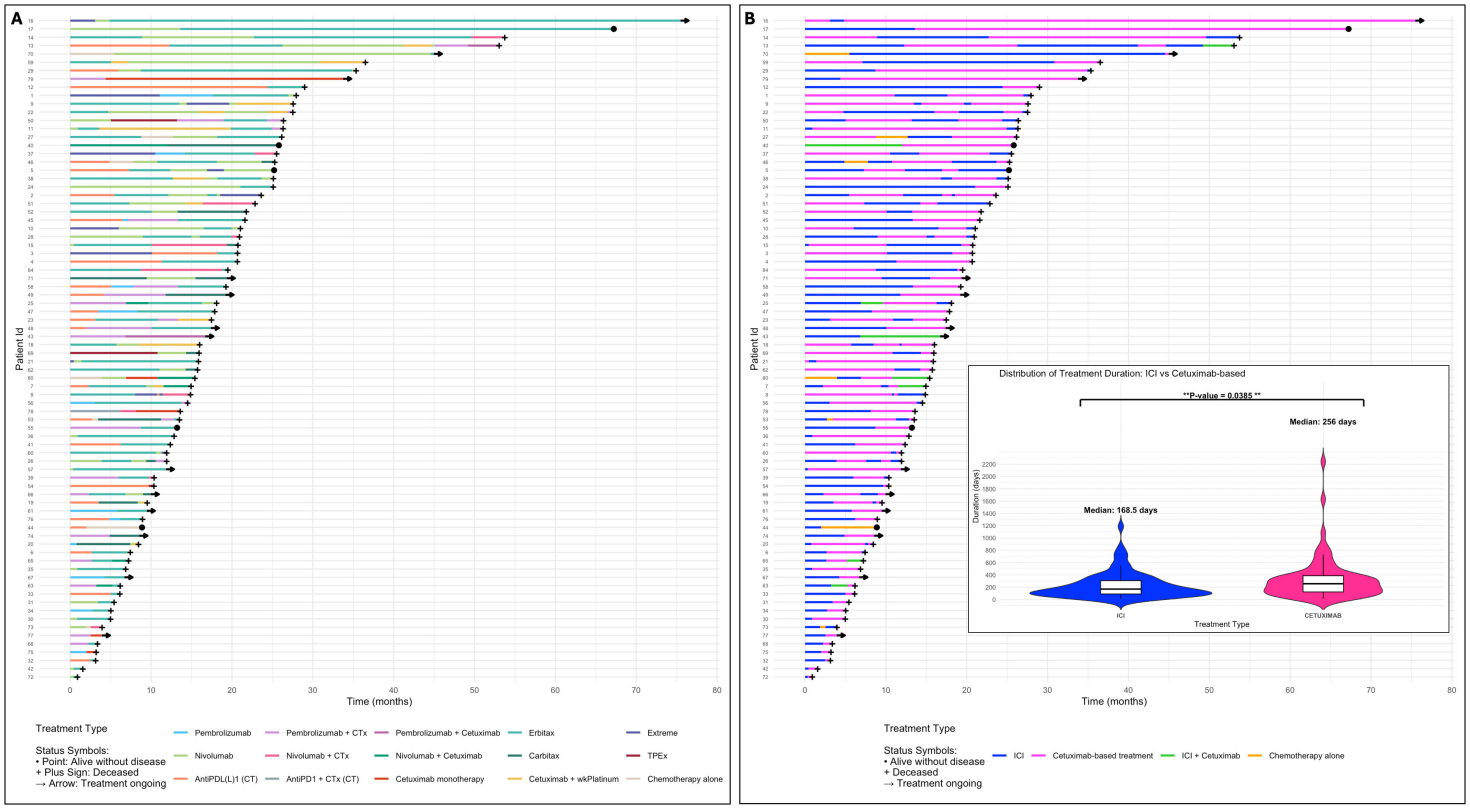
Swimmer plots illustrating the type and duration of each line of systemic treatment in the R/M setting for the whole population. On the left, specific type of systemic therapy **(A)**. On the right **(B)**, the same plot indicates the systemic therapy group administered during each line of therapy. The violin plots depict the comparison of treatment duration after encompassing all lines that included ICI and all lines including cetuximab. Lines combining ICI and cetuximab and those consisting of chemotherapy alone were not included in final sum of ICI or cetuximab-based treatment periods. For more details, please refer to the main text. Carbitax, cetuximab + weekly paclitaxel + weekly carboplatin; (CT), clinical trial; CTx, chemotherapy; Erbitax, cetuximab + weekly paclitaxel; Extreme, cetuximab + platinum + 5-FU; ICI, immune checkpoint inhibitor; TPEx, cetuximab + docetaxel + platinum; wkPlatinum, weekly platinum; R/M, recurrent/metastatic; 5-FU, 5-fluorouracil.

Among the 80 patients, treatment durations were categorized into two groups: ICI-based therapy and non-ICI cetuximab-based therapy. Treatment periods involving chemotherapy alone or anti-PD-1 plus anti-EGFR combinations were excluded from the analysis. The median duration of cetuximab-based therapy was significantly longer than that of ICI therapy (256 *vs*. 168.5 days; p = 0.0385). This difference was maintained in favor of cetuximab for patients treated with first SCAI in second line (157.5 *vs*. 134.5 days, p = 0.55) but was only statistically significant for patients treated with first SCAI as third or fourth line (360.5 *vs*. 237.5 days, p = 0.009) (**Figure 3**, **Supplementary Figure S12**). No differences were observed in the duration of first or second SCAI compared to that of immediately prior ICI therapy (**Supplementary Figure S12**).

### Safety

All patients experienced grade 1–2 adverse events (AEs) during both the first and second SCAI. Grade 3–4 AEs occurred in 38.7% of patients during first SCAI and 47% during second SCAI, with no toxicity-related deaths. For first SCAI, the most common grade 3–4 AEs were neutropenia (12.5%), pneumonia (8.8%), rash (6.3%), and tumor bleeding (6.3%, n = 5, all grade 3). One patient died due to respiratory sepsis after bronchoaspiration pneumonia during first SCAI, which was considered unrelated to treatment. For second SCAI, the most common grade 3–4 AEs were pneumonia (29.4%, n = 5, all grade 3), anemia (11.8%), neutropenia (11.8%), and sepsis (11.8%). No new safety signals were identified compared to the known safety profiles of the regimens used. Safety outcomes are summarized in **Supplementary Table S4**.

## Discussion

In this updated series of 80 patients with R/M SCCHN, first SCAI achieved an ORR of 59%. First SCAI was administered as second-line in 60% of patients and as third- or fourth-line in 40%. Median PFS and OS since first SCAI initiation were 5.9 and 12.4 months, respectively. The most common regimen, Erbitax (weekly cetuximab plus paclitaxel), used in 64% of first SCAI cases, yielded an ORR of 63%, a median PFS of 6 months, and an OS of 12.4 months. Responses were profound, with a median PCTL of −57% in both the total population and Erbitax-treated patients. In the Keynote-048 study, among the patients receiving SCAI after progression to ICI, PFS2 was significantly longer for those receiving taxanes after pembrolizumab plus platinum–5-fluorouracil (5-FU) (5). Kacew et al. (6) found comparable ORR, PFS, and OS for taxane-based and cetuximab-based SCAI. However, Watanabe et al. (11) reported an ORR of 54.9% with cetuximab plus paclitaxel (n = 72), compared to 27.9% for taxane monotherapy (n = 89) and 25.5% for tegafur/gimeracil/oteracil (S1) (n = 48), with a median PFS and OS of 5.4 and 13 months, respectively, in the whole population. Likewise, Llop et al. (10) reported a 49% ORR and a median PFS and OS of 4.1 and 9.3 months, respectively, in 63 heavily pretreated patients receiving SCAI ( ± cetuximab), with higher ORR, PFS, and OS with Erbitax compared to paclitaxel monotherapy. Koyama et al. (12) reported in a phase II non-randomized study of 35 patients pretreated with anti-PD1 and platinum, who, after progression, received biweekly cetuximab plus paclitaxel (100 mg/m^2^, d1, 8, and 15) followed by maintenance biweekly cetuximab, an ORR of 69.6% and a median PFS and OS of 5.5 and 13.3 months, respectively. Tanaka et al. (13) found a 62.7% ORR and a median PFS and OS of 4.6 and 17.1 months, respectively, in 59 patients treated with cetuximab plus paclitaxel ± carboplatin. Saleh et al. (7) observed a 50% ORR with cetuximab-based SCAI *vs*. 30% overall in 82 heavily pretreated patients, with a median PFS and OS of 3.6 and 7.8 months, respectively. Finally, Fuereder et al. (15) reported in a recent phase II study of 57 patients receiving three-weekly paclitaxel plus weekly cetuximab for six cycles, followed by maintenance cetuximab after progression to ICI, an ORR reaching 47.4% with 14% of complete responses, and the median DoR, PFS, and OS achieved since SCAI initiation were 5.5, 5.9, and 14 months, respectively. Thus, our findings not only confirm our previously published data but also align with recent retrospective series and non-randomized trials reporting high ORR and survival outcomes with cetuximab-based and taxane-based SCAI (5–15, 22). Our series and the aforementioned studies contrast with the existing data from the pre-ICI era for second-line cetuximab-based chemotherapy, reporting an ORR of 13%–35% and a median PFS of 2–4 months and OS of 5.5–7.5 months (6–9). In this regard, while the ORR of second-line cetuximab monotherapy pre-ICI was 13%, post-ICI ORR of between 23.9% and 37.5% has been reported in recent years (23–25). Interestingly, one of our patients (Id 79) achieved a complete response with second-line cetuximab + monalizumab after progression to pembrolizumab, carboplatin, and paclitaxel, which has been ongoing for more than 30 months (**Supplementary Figure S10**). These findings suggest that the efficacy of single-agent cetuximab can also be boosted by prior ICI and may be a promising post-ICI chemo-free therapeutic option.

To the best of our knowledge, with 77 of 80 patients receiving cetuximab-based SCAI [the remaining three patients (Id 44, 46, and 73) received post-ICI taxane + platinum-based chemotherapy without cetuximab], our series is the largest one published to date incorporating cetuximab into SCAI and reinforces the important role of anti-EGFR agents in the post-ICI setting, particularly when combined with taxanes.

In 22 of the patients who had received chemotherapy before (LCBI) and after (SCAI) immunotherapy, we demonstrate that the ORR was higher with SCAI than with LCBI (47% *vs*. 36%). In this subpopulation, the median PFS was, however, longer with LCBI than with SCAI (8 *vs*. 4.4 months). These findings indicate that ICI treatment may potentiate the response to subsequent SCAI. The shorter PFS of SCAI compared to LCBI may be explained by the more heavily pretreated nature of the patients during SCAI, although a component of pseudoprogression to ICI that magnifies the response to SCAI without an impact on PFS cannot be disregarded. Anyhow, the median OS since the first SCAI reached 9.4 months, which is notable considering that these patients were at least in a third line of treatment. Indeed, the OS of these 22 LCBI-treated patients since the start of first-line therapy was 25.9 months, in line with the OS of the whole series (24 months) and more than doubling the OS in the total population of any of the arms of the Keynote-048 trial (1).

We also evaluated the performance of a second SCAI in a subgroup of 17 patients who were rechallenged with ICI and subsequently treated with SCAI. The ORR reached 30%; the median PFS and OS since second SCAI were 3.5 and 7.1 months, respectively; and OS since first line was 21.7 months, which are notable considering the very heavily pretreated nature of these patients, suggesting that rechallenging patients with ICI may boost responses and enable a longer disease control with a second line of SCAI in selected patients (i.e., see Id 5 in **Figure 3** and **Supplementary Figure S11**). It is interesting to note that all patients had progressed radiologically and symptomatically to prior cetuximab, prior ICI and to first SCAI, so all these patients were deemed cetuximab- and ICI-refractory. Among these patients, only one patient showed a partial response to ICI rechallenge (Id 2) (24).

In the total population of 80 patients with R/M SCCHN, median OS since first-line initiation was 24 months. No differences in OS were observed by PD-L1 expression (CPS <20 *vs*. ≥20; p = 0.97), suggesting that the predictive effect of PD-L1 may be diminished in cohorts receiving multiple treatment lines. However, OS was numerically longer in patients with CPS ≥ 20, indicating that heterogeneity and limited sample size may have obscured a clear OS difference between high and low PD-L1 expression. Prior studies have reported conflicting results on the impact of PD-L1 expression. Llop et al. (10) identified PD-L1 expression as an independent risk factor for ORR, PFS, and OS, whereas Tanaka et al. (13) found no association with PFS or OS. We also evaluated OS depending on the treatment line of the first SCAI and found a numerically longer OS for patients treated in the ≥3rd line compared to the 2nd line (26.2 *vs*. 16.6 months, p = 0.16). While not statistically significant, this difference, if real, may simply indicate a potential immortality bias favoring those patients naturally selected to perform better. However, this finding could also indicate a possible difference in efficacy depending on the treatment sequence used, which should be explored in randomized studies, given the absence of data available to date.

We analyzed treatment exposure from first-line therapy, categorizing therapeutic periods into ICI-based and cetuximab-based therapies. The median duration of cetuximab-based therapy was significantly longer than ICI therapy (256 *vs*. 168.5 days; p = 0.038), indicating that anti-EGFR agents are critical in the R/M setting, with activity potentially enhanced by prior ICI exposure. As expected, this difference was particularly notable for patients treated with 1st SCAI in the ≥3rd line, as most of these patients had started a cetuximab-based first line of therapy prior to pre-SCAI ICI. Our findings indicate that cetuximab-based therapy, particularly when combined with taxanes, should be offered after rapid or symptomatic progression on ICI, as it may achieve durable tumor reduction and improve survival compared to historical pre-ICI data, even in cetuximab pre-treated patients. Indeed, in our series, 20 patients treated with cetuximab-based LCBI received cetuximab-based first SCAI and among the 17 patients treated with second SCAI -in all cases 2nd SCAI was cetuximab-based-, all had progressed to prior-line cetuximab (6–9).

As a whole, the data presented in our study not only align with those of prior studies informing of the enhanced activity and potentially increased efficacy of cetuximab-based treatments post-ICI but may also lead to a new issue, which is the role of sequential treatments, including ICI- and anti-EGFR rechallenge, and may open the possibility for individualized sequential treatment strategies in patients with R/M SCCHN (26).

The safety profile during SCAI was consistent with expectations, showing no new safety signals compared to pre-ICI era profiles. While five patients with bulky disease after ICI progression experienced grade 3 tumor bleeding during first SCAI, likely due to rapid and profound tumor shrinkage, tumor bleeding is a common occurrence in advanced disease settings, and therefore, a direct pro-hemorrhagic effect of SCAI was disregarded. However, clinicians should be aware of this potential complication and educate patients with high tumor volume or with tumors close to large vessels or under anticoagulants and, in extremely high-risk patients, even consider an in-hospital observation period for a few days at the start of SCAI.

Our study has several limitations. Despite being one of the largest series published to date on the role of SCAI in R/M SCCHN, the sample size is limited, and our population is heterogeneous, with 60% of patients treated in second line and the remaining in the third or fourth line. The expression of PD-L1 measured by CPS showed a wide range, but the median CPS was 20, thereby in the high range. However, this was only slightly higher than the median CPS in a large phase 3 study such as Keynote-048 (1). The percentage of first-line platinum refractory patients (14%) was also within the expected range in the literature (2). Finally, in our series, 42.5% of patients received at least one line of therapy post-first SCAI, 39% were rechallenged with ICI post-SCAI, and in up to 17 patients, a second SCAI was even offered. Therefore, the median number of lines of therapy was higher than that in referent randomized phase III studies with ICIs published to date in the R/M setting (1, 2, 25–28). This may explain the longer OS since the first line in our series, but could limit the generalizability of our findings.

## Conclusion

Sequential cetuximab-based salvage therapy after ICIs demonstrated high ORR, profound and durable responses, and improved survival compared to historical pre-ICI data. These results should be confirmed in a larger prospective study.

## Data Availability

The raw data supporting the conclusions of this article will be made available by the authors, without undue reservation.

## Glossary

AE(s): adverse event(s)
BOR: best objective response
CI: confidence interval
CPS: combined positive score
CR: complete response
CRT: chemoradiotherapy
CTx: chemotherapy
CT: clinical trial
ECOG PS: Eastern Cooperative Oncology Group Performance Status
ERBITAX: cetuximab + weekly paclitaxel
EXTREME: cetuximab + three-weekly platinum + 5-fluorouracil
G1-2: grade 1–2
G3-5: grade 3–5
Gy: gray
HPV: human papillomavirus
HR: hazard ratio
HRF: pathological high-risk factors
HypoMg++: hypomagnesemia
ICI: immune checkpoint inhibitor
LCBI: last chemotherapy before immunotherapy
LRTI: lower respiratory tract infection
m: months
Multi: multivariate analysis
NIVO: nivolumab
OPC: oropharyngeal cancer
ORR: objective response rate
OS: overall survival
PD: progressive disease
PD1: programmed-death receptor 1
PD-L1: programmed-death receptor 1 ligand
PEMBRO: pembrolizumab
PFS: progression-free survival
Pop: population
PR: partial response
RECIST 1.1: Response Evaluation Criteria In Solid Tumors version 1.1
RT: radiotherapy
R0: macro- and microscopically complete resection
R/M: recurrent/metastatic
SCAI: salvage chemotherapy after immunotherapy
SCC: squamous cell carcinoma
SCCHN: squamous cell carcinoma of the head and neck
SD: stable disease
TL: target lesion
TPEx: cetuximab + platinum + docetaxel
TPF: platinum + 5-fluorouracil + docetaxel
Uni: univariate analysis
URTI: upper respiratory tract infection
wkCarbo: weekly carboplatin
wkCDDP: weekly cisplatin
3wkCarbo: three-weekly carboplatin
3wkCDDP: three-weekly cisplatin
wkPlatinum: weekly platinum
