# Supplementary material for: Sequential salvage systemic therapy after immunotherapy in head and neck cancer: a real-world study
Source: Front Oncol. 2026 Jan 9;15:1719793. doi: 10.3389/fonc.2025.1719793 (PMC12827138; doi:10.3389/fonc.2025.1719793)
Supplement: Supplementary file 1 [file DataSheet1.docx]

**SUPPLEMENTARY MATERIAL**

**FIGURES S1 – S12:** Pages 2-21

**TABLES S1 – S4:** Pages 22 - 25

**FIGURES**

**Figure S1.** Flowchart illustrating the patient allocation process. Patients were included in the study if they had received at least a first SCAI (N=80), regardless of the number of prior lines of therapy within the recurrent/metastatic setting. Among these 80 patients, LCBI had been administered in 22 patients, and a 2^nd^ SCAI was administered in 17 patients. ICI: immune checkpoint inhibitors, LCBI: last chemotherapy before immunotherapy, SCAI: salvage chemotherapy after immunotherapy, SCCHN: squamous cell carcinoma of the head and neck.

**
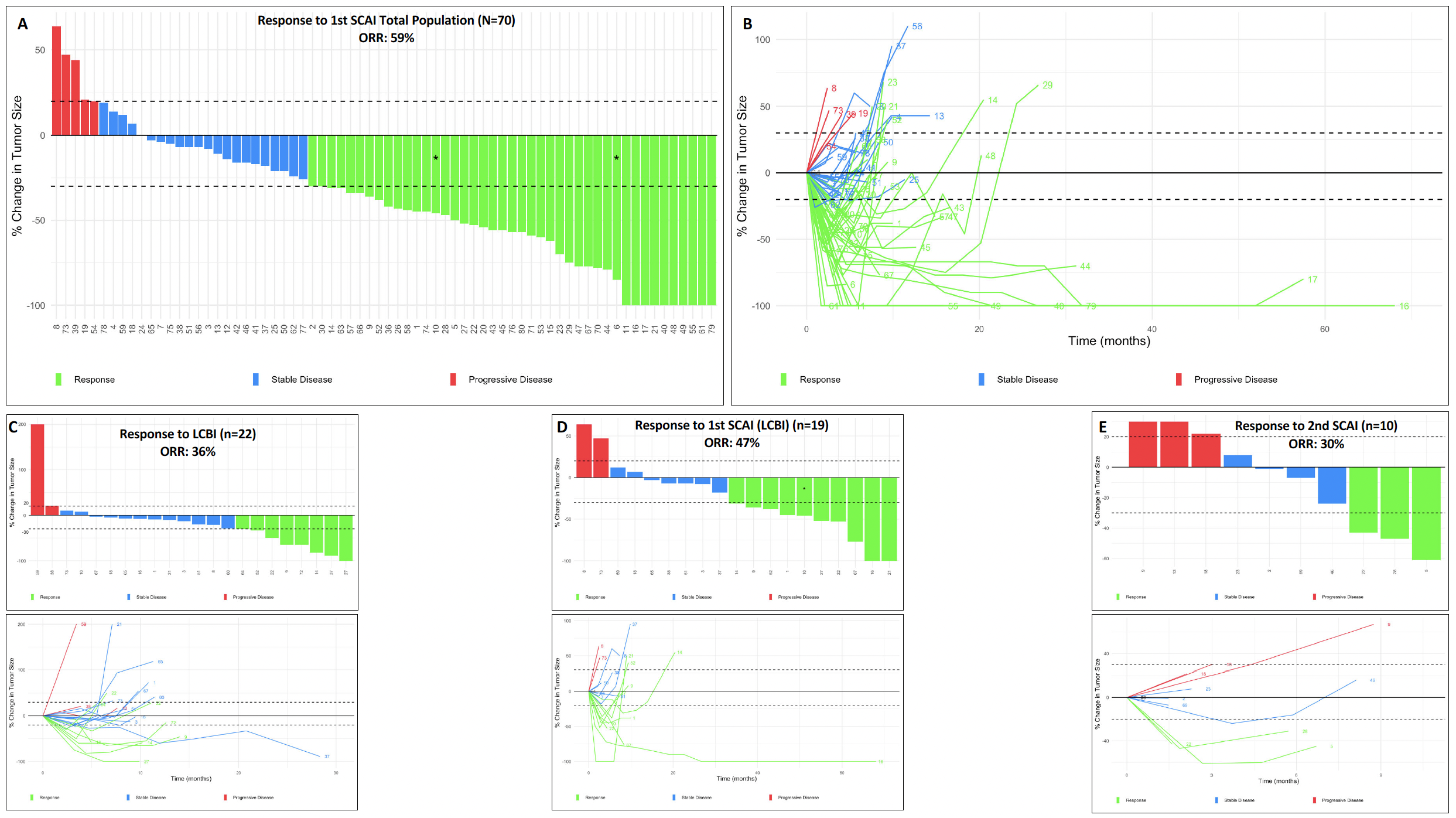
**

**Figure S2.** Waterfall and spider plots depicting the response to 1^st^ SCAI, LCBI, 1^st^ SCAI in LCBI treated patients, and to 2^nd^ SCAI. Type of response achieved in target lesions with 1^st^ SCAI (A,B), LCBI (C), 1^st^ SCAI (LCBI treated) (D), and 2^nd^ SCAI (E). LCBI: last chemotherapy before immunotherapy; ORR: objective response rate; SCAI: salvage chemotherapy after immunotherapy. Asterisks (*) indicate patients (Id 6 and 10) who achieved and overall progressive disease, despite responding in target lesions, due to the appearance of new non-measurable (bone) metastatic lesions. For more details, please refer to the main text.

**
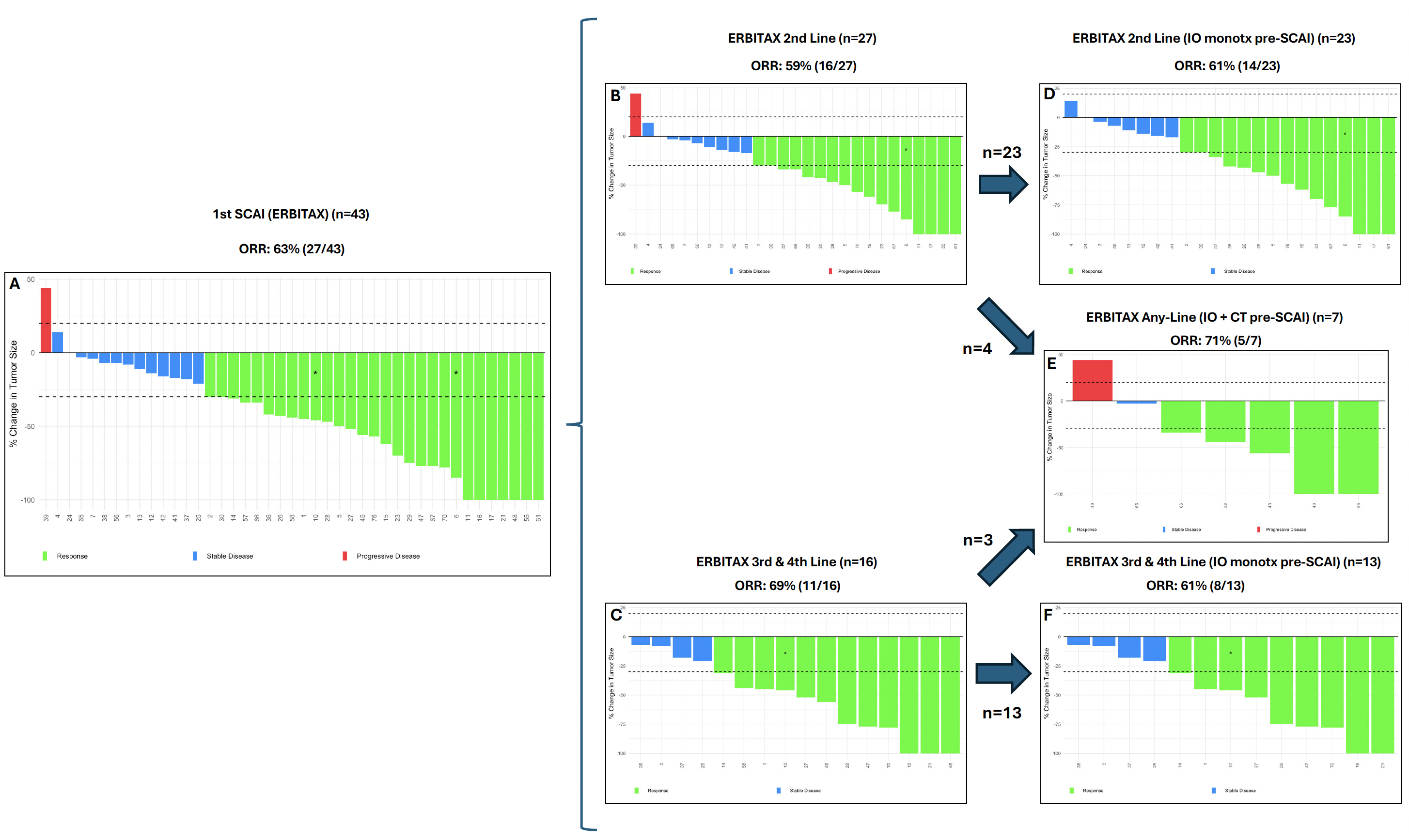
**

**Figure S3.** Waterfall plots depicting the objective response rate in target lesions to 1^st^ SCAI with Erbitax. ORR in TL in all patients treated with Erbitax as 1^st^ SCAI (A), when used in 2^nd^ line (B), in 3^rd^ and 4^th^ lines (C) and when used after ICI monotherapy (D,F) and after ICI + CT (E) as pre-SCAI. Erbitax: weekly cetuximab plus paclitaxel; ORR in TL: objective response rate in target lesions; SCAI: salvage chemotherapy after immunotherapy. Asterisks (*) indicate patients who achieved an overall progressive disease, despite responding in target lesions, due to the appearance of new non-measurable (bone) metastatic lesions. For more details, please refer to the main text.


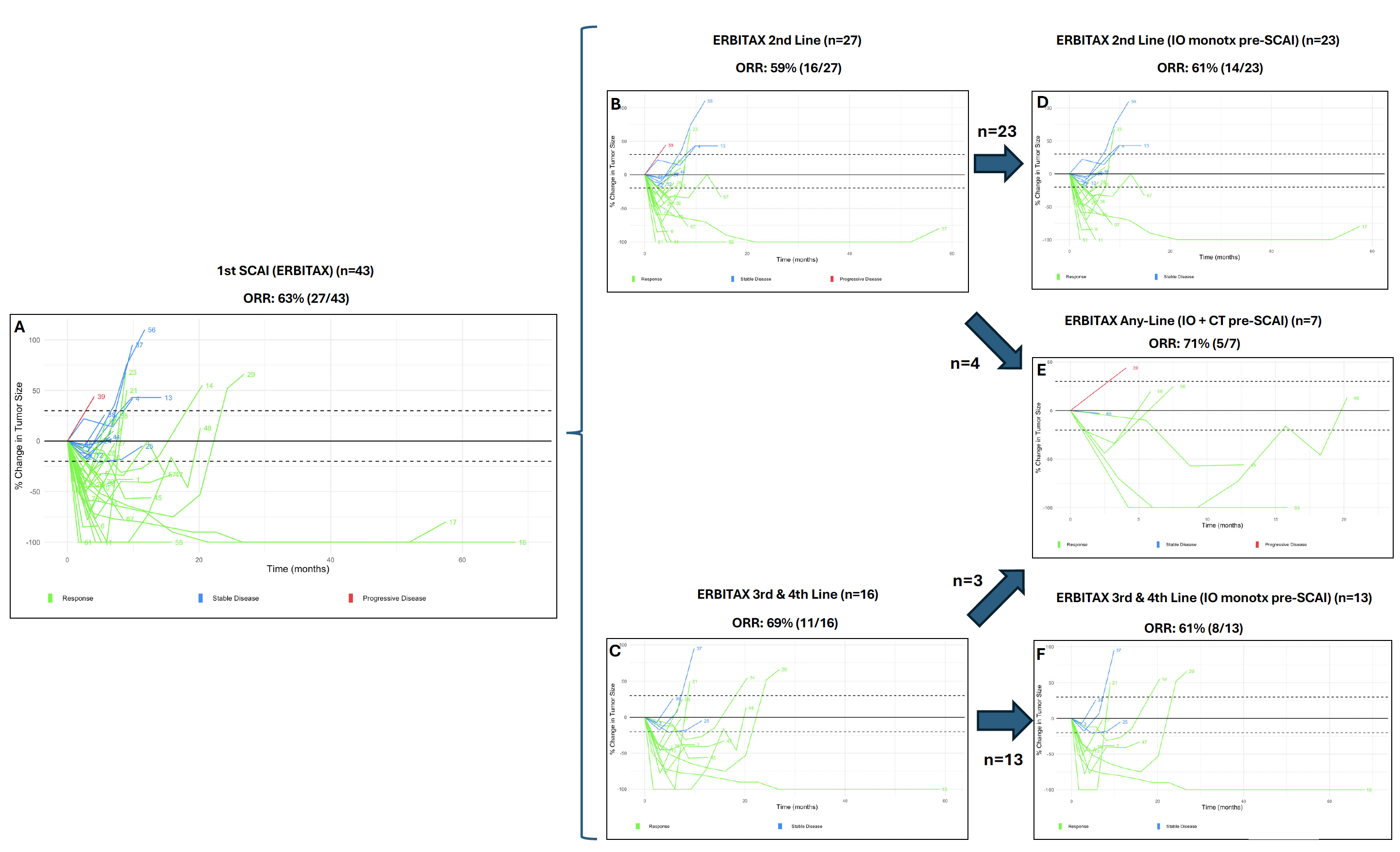


**Figure S4.** Spider plots depicting the objective response rate in target lesions to 1^st^ SCAI with Erbitax. ORR in TL in all patients treated with Erbitax as 1^st^ SCAI (A), when used in 2^nd^ line (B), in 3^rd^ and 4^th^ lines (C) and when used after ICI monotherapy (D,F) and after ICI + CT (E) as pre-SCAI. Erbitax: weekly cetuximab plus paclitaxel; ORR in TL: objective response rate in target lesions; SCAI: salvage chemotherapy after immunotherapy. Cases 6 and 10 achieved an overall progressive disease, despite responding in target lesions, due to the appearance of new non-measurable (bone) metastatic lesions. For more details, please refer to the main text.


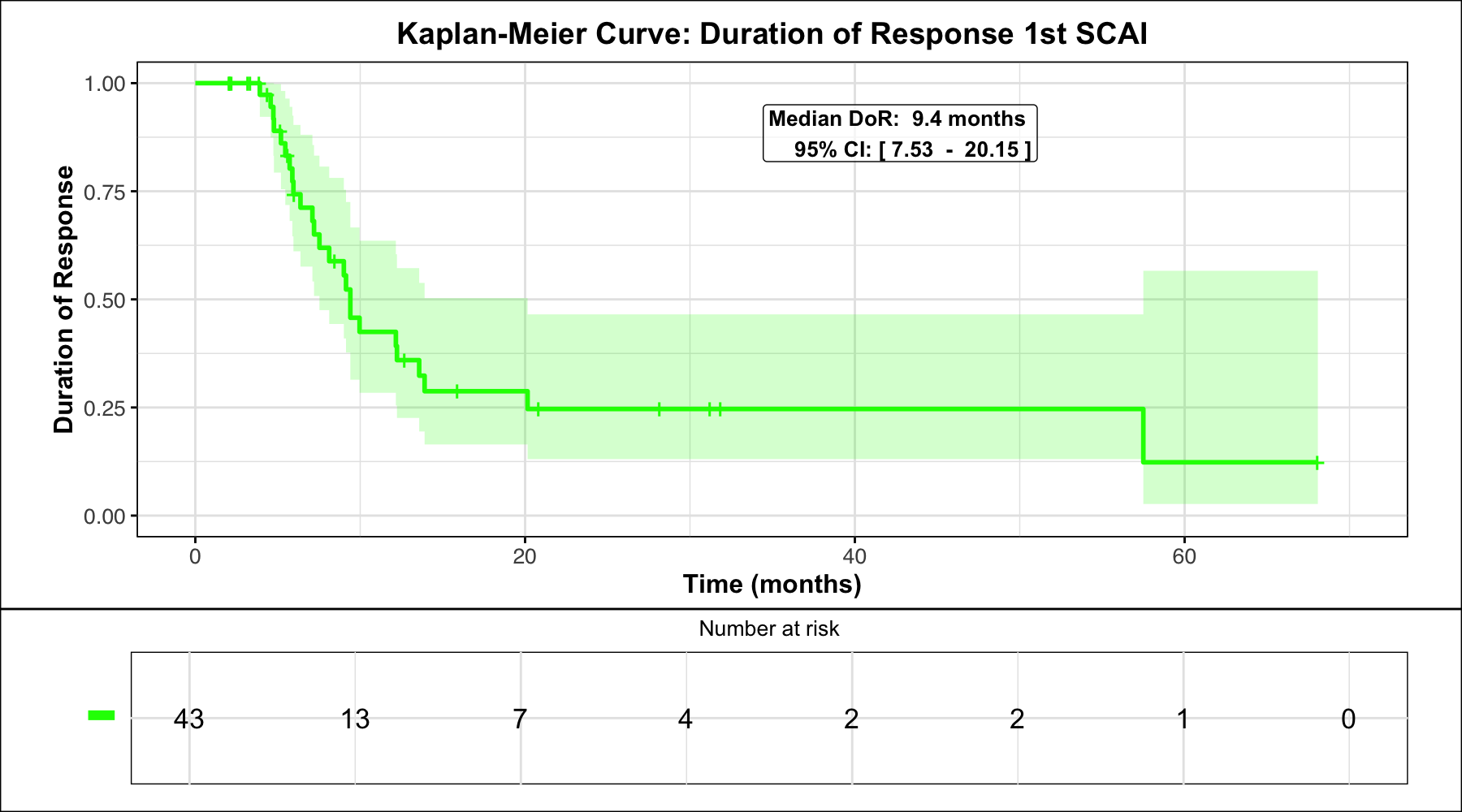

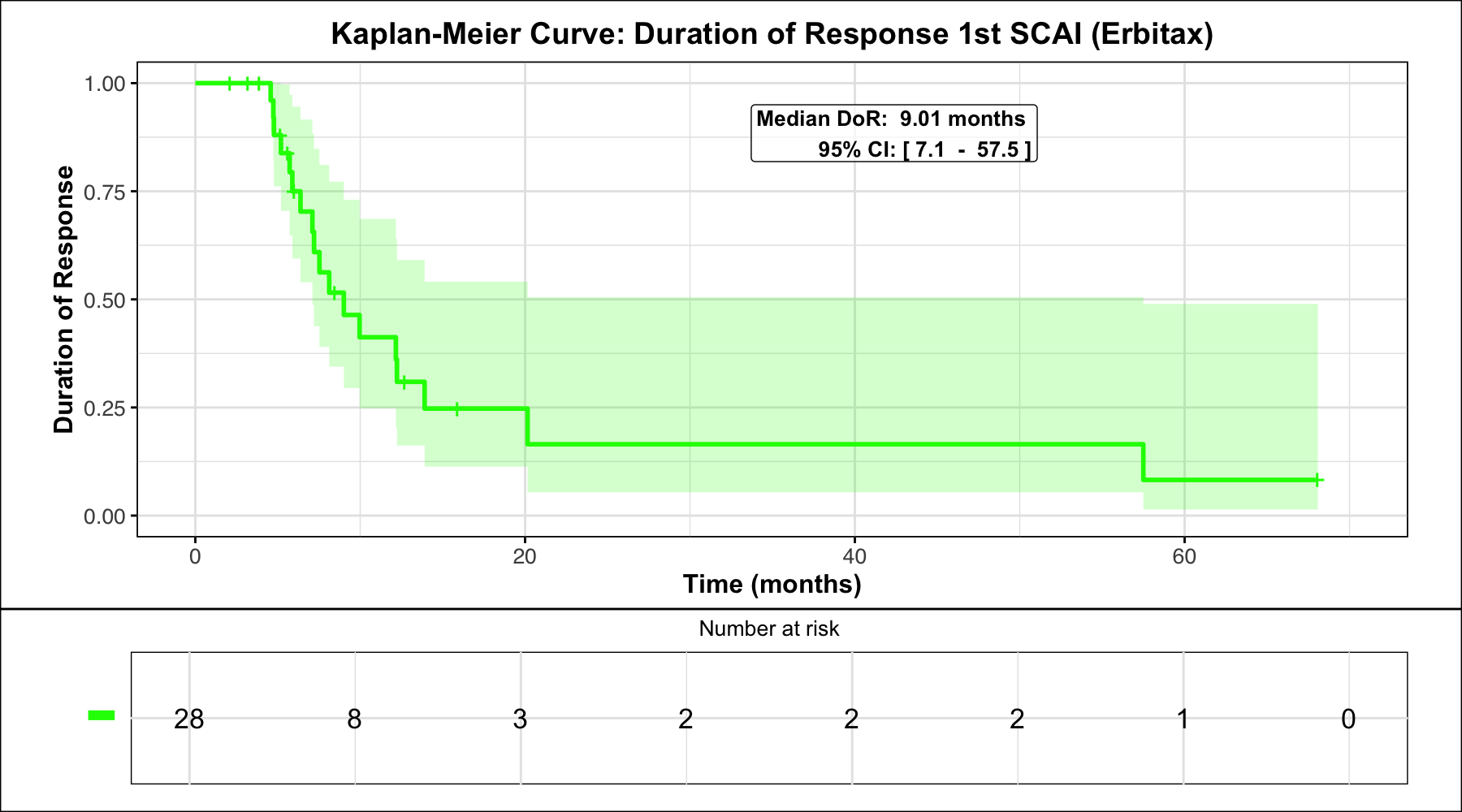


**A**

**B**

**Figure S5.** Duration of response during 1^st^ SCAI in the total population (A) and in patients treated with Erbitax (B).

Erbitax: weekly cetuximab plus paclitaxel; SCAI: salvage chemotherapy after immunotherapy.


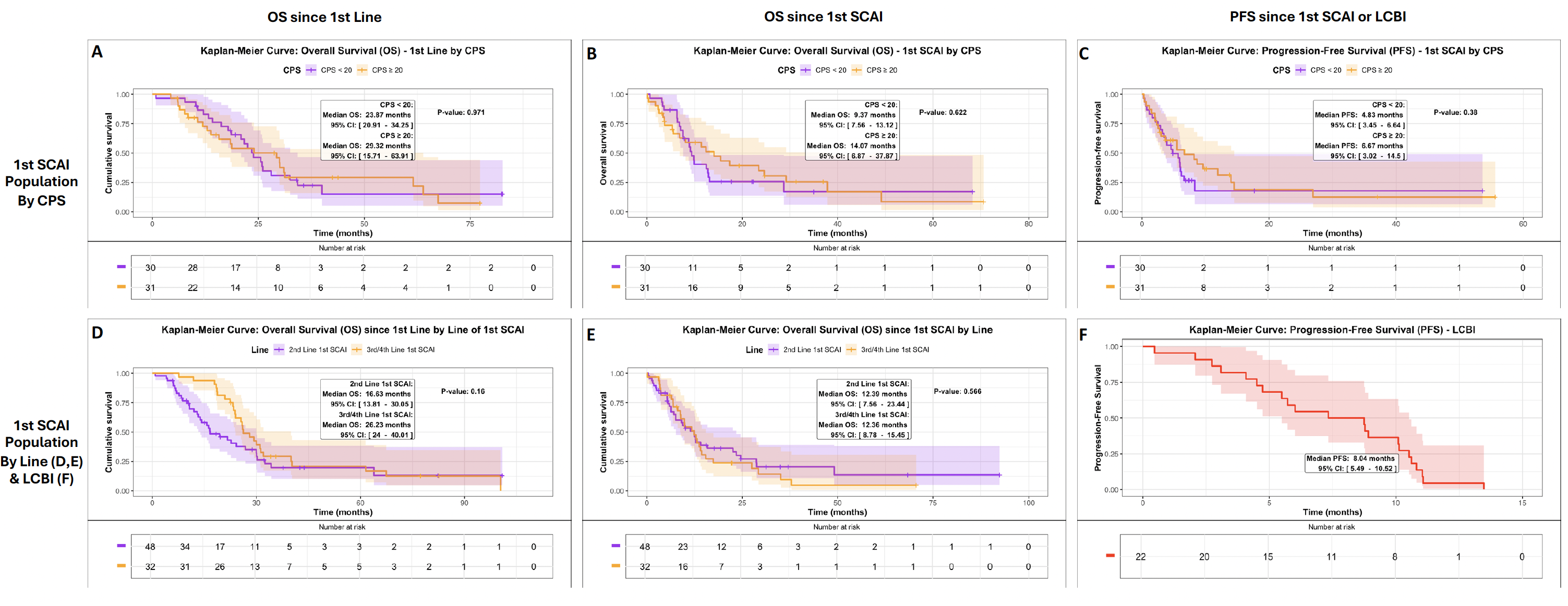


**Figure S6.** Survival of patients treated with 1^st^ SCAI by CPS and by treatment line and during LCBI. OS since 1^st^ line by CPS (A) and by treatment line (D). OS since 1^st^ SCAI by CPS (B) and by treatment line (E). and according to PDL1 expression (A-D). PFS since 1^st^ SCAI according to PDL1 expression (C), and PFS during LCBI (F). CPS: combined positive score, LCBI: last chemotherapy before immunotherapy, PFS: progression-free survival, OS: overall survival, SCAI: salvage chemotherapy after immunotherapy.

**
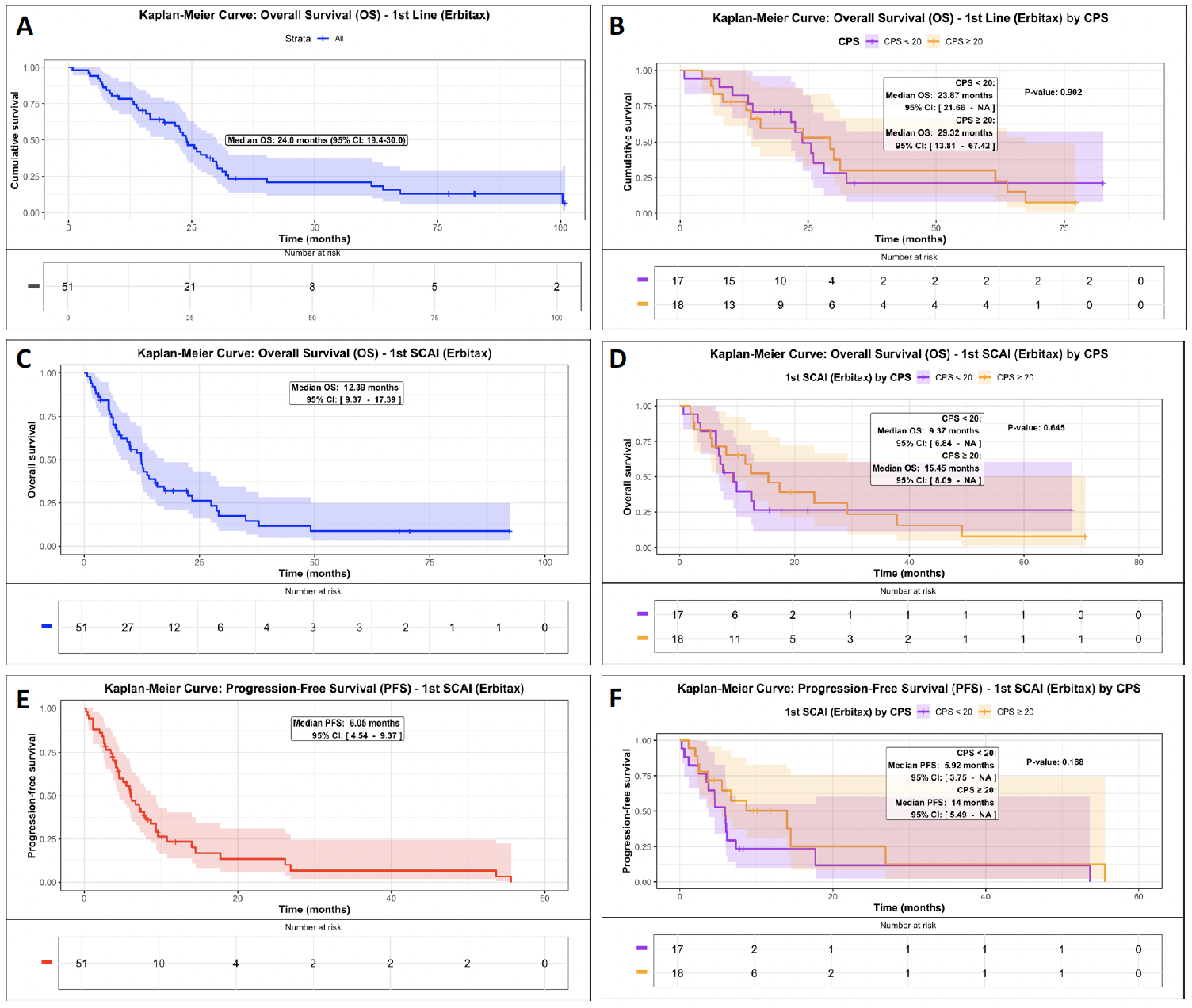
**

**Figure S7.** Survival of patients treated with Erbitax as 1^st^ SCAI. Overall survival (OS) since 1^st^ line and since 1^st^ SCAI and according to PDL1 expression (A-D). PFS since 1^st^ SCAI and according to PDL1 expression (E,F). CPS: combined positive score, Erbitax: weekly cetuximab plus paclitaxel; PFS: progression-free survival, SCAI: salvage chemotherapy after immunotherapy.

**
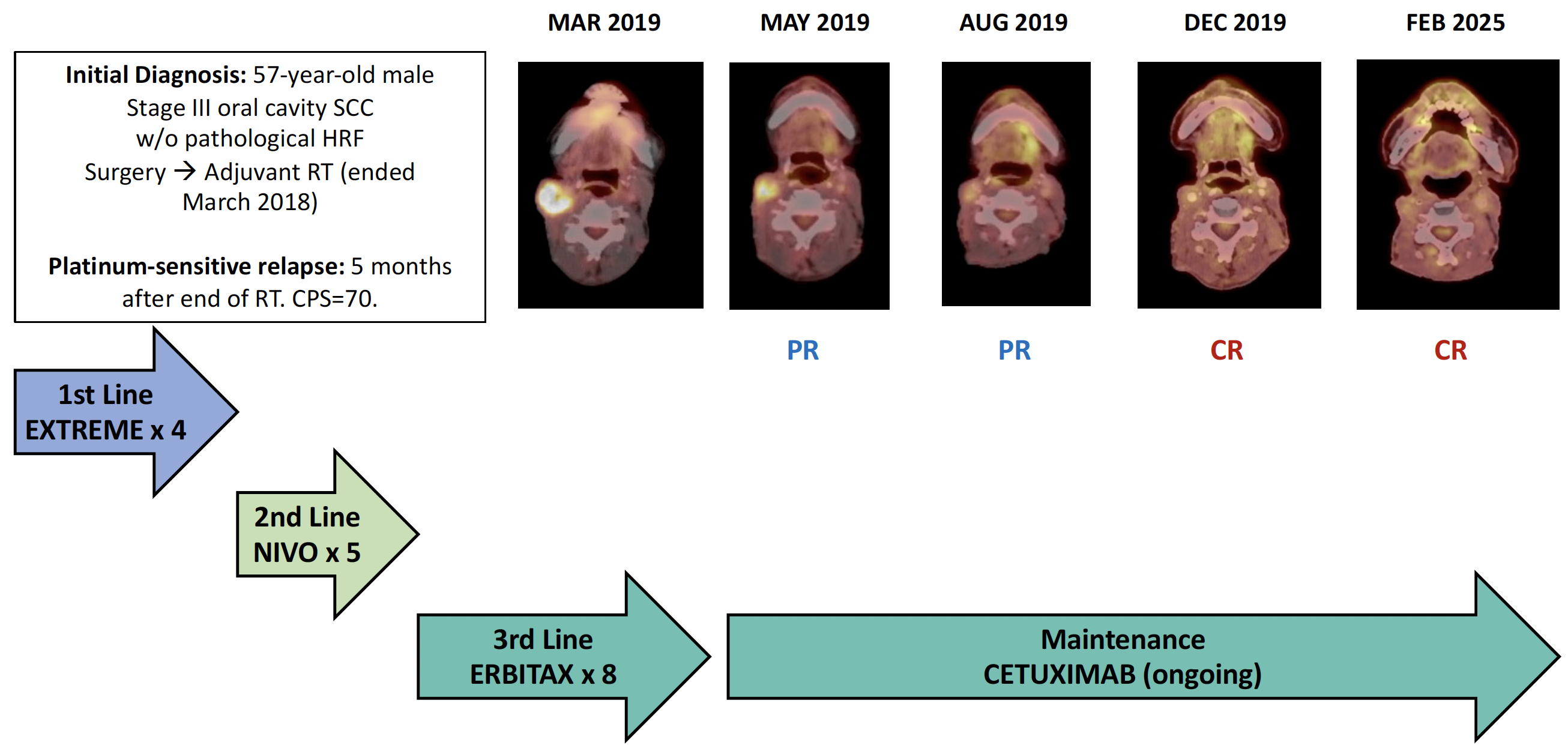
**

**Figure S8.** Case example of a patient (**Id 16**) treated with 3^rd^ line SCAI with Erbitax after a rapid and symptomatic progression to 2^nd^ line nivolumab. As shown in the figure, the patient is currently under reduced-dose cetuximab maintenance (300 mg/m2 every 3 weeks due to prior grade 3 rash and mucositis, that improved to grade 1) and has been disease-free for 60 months. There is no established time-limit for duration of cetuximab treatment, as far as continued clinical benefit without limiting toxicity are present. CPS: combined positive score, CR: complete response, Erbitax: weekly paclitaxel plus cetuximab, Extreme: cetuximab plus platinum-5FU, Nivo: nivolumab, PR: partial response, RT: radiotherapy, SCC: squamous cell carcinoma.


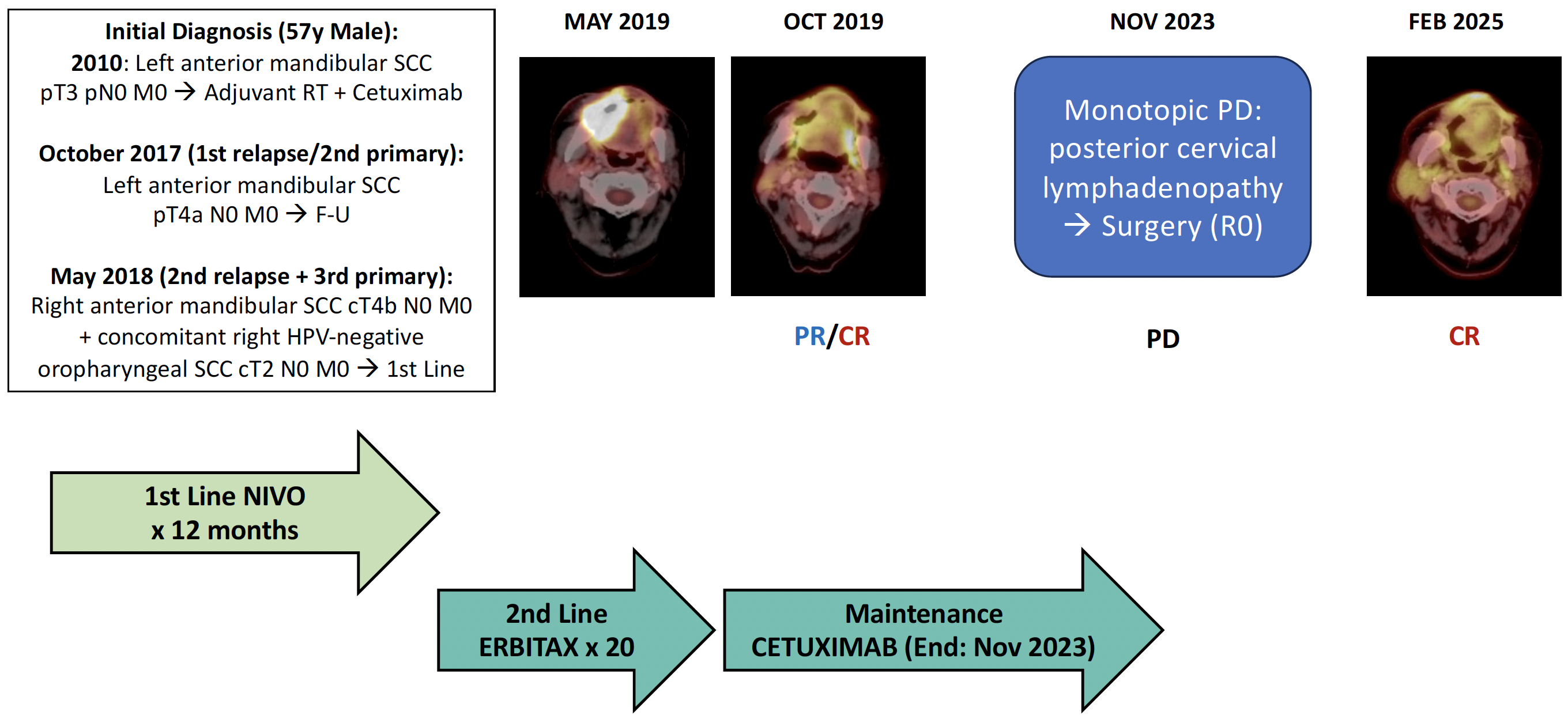


**Figure S9.** Case example of a patient (**Id 17**) treated with 2^nd^ line SCAI with Erbitax after a symptomatic progression to 1 year of therapy with 1^st^ line nivolumab. As shown in the figure, the patient achieved a probable complete response after 20 weeks of Erbitax, continuing thereafter with maintenance biweekly cetuximab. Complete response continued until November 2023, when a monotopic posterior cervical lymphadenopathy occurred that was completely resected. The patient continued follow up with no signs of relapse at last follow-up, having achieved an OS of 69 months since the start of 2^nd^ line Erbitax post-ICI. CR: complete response, Erbitax: weekly paclitaxel plus cetuximab, NIVO: nivolumab, PD: progressive disease, PR: partial response, R0: complete and margin-negative resection, SCC: squamous cell carcinoma.


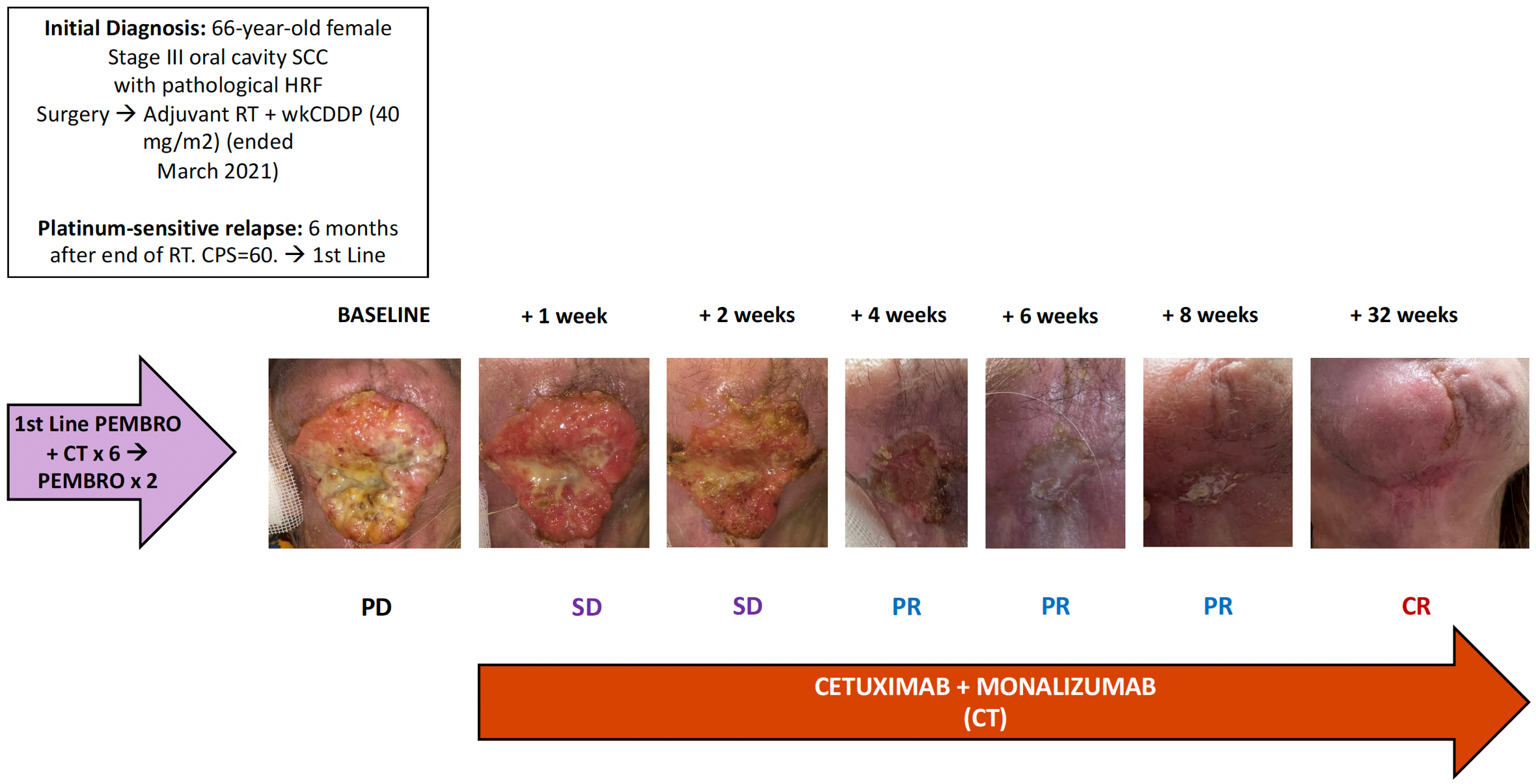


**Figure S10.** Case example of a patient (**Id 79**) treated with 2^nd^ line Cetuximab + Monalizumab within a clinical trial (NCT04590963. AstraZeneca, the trial sponsor allowed for the publication of the data and the patient consented for the publication of the images), after a symptomatic progression to 1^st^ line pembrolizumab + 3wkCarboplatin + Paclitaxel. As shown in the figure, the patient achieved a probable complete response after 32 weeks of treatment. The patient continued with cetuximab + monalizumab until study closure in 2023, continuing thereafter with cetuximab maintenance, until May 2025, in which it was decided to be terminated 38 months after starting 2^nd^ line therapy. CPS: combined positive score, CR: complete response, (CT): clinical trial, HRF: pathological high-risk factors, PEMBRO: pembrolizumab, PD: progressive disease, PR: partial response, RT: radiotherapy, SCC: squamous cell carcinoma, SD: stable disease, wkCDDP: weekly cisplatin.


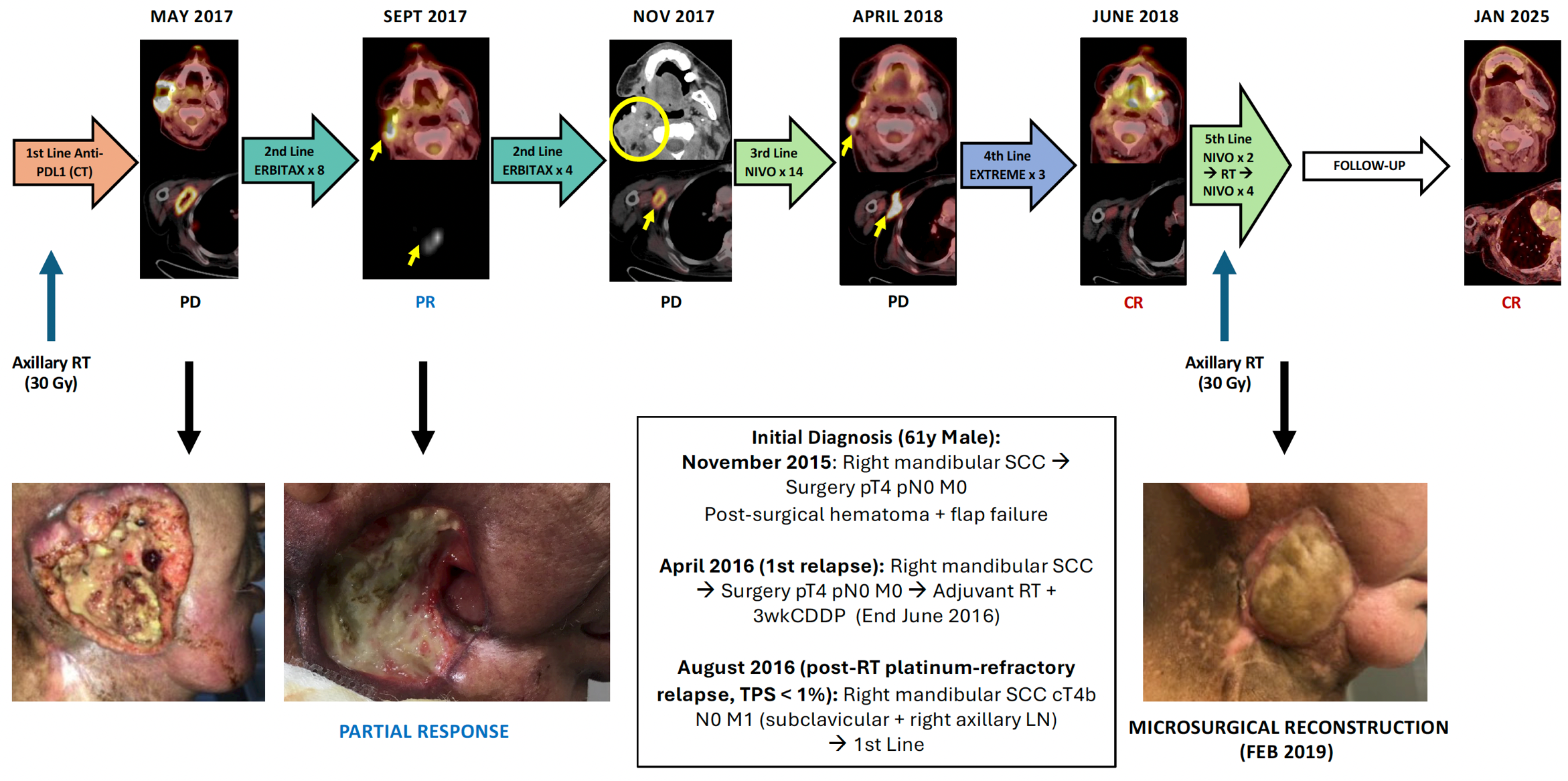


**Figure S11.** Case example of a patient (**Id 5**) treated with 1^st^ and 2^nd^ SCAI. 2^nd^ line SCAI with Erbitax after a symptomatic progression to prior therapy with anti-PDL1 within a clinical trial. As shown in the figure, the patient showed a large, ulcerated lesion of 5 cm in the right cheek communicating with the oral cavity. Three months after starting 1^st^ SCAI with Erbitax, a partial response had been achieved. Progressive disease occurred 6 months after starting Erbitax, and the patient was rechallenged with ICI (nivolumab) that the patient received for 6 months. Subsequently the patient started 2^nd^ SCAI with reduced-dose Extreme (25% dose reduction of cisplatin and 5FU, with full dose cetuximab) achieving a complete response. Due to re-apperance of a tumor ulceration (histologically confirmed) in the right axilla, 5^th^ line nivolumab was administered for 2 doses, followed by consolidation hypofractionated RT (30 Gy) over the right axillary region and continuing thereafter with nivolumab for 4 additional doses until December 2018, achieving a complete response, still ongoing at data cut-off (February 2025), 81 months after starting 2^nd^ SCAI. CR: complete response, ERBITAX: weekly paclitaxel plus cetuximab, EXTREME: three-weekly platinum-5FU plus cetuximab, NIVO: nivolumab, PD: progressive disease, PR: partial response, R0: complete and margin-negative resection, SCC: squamous cell carcinoma.

**
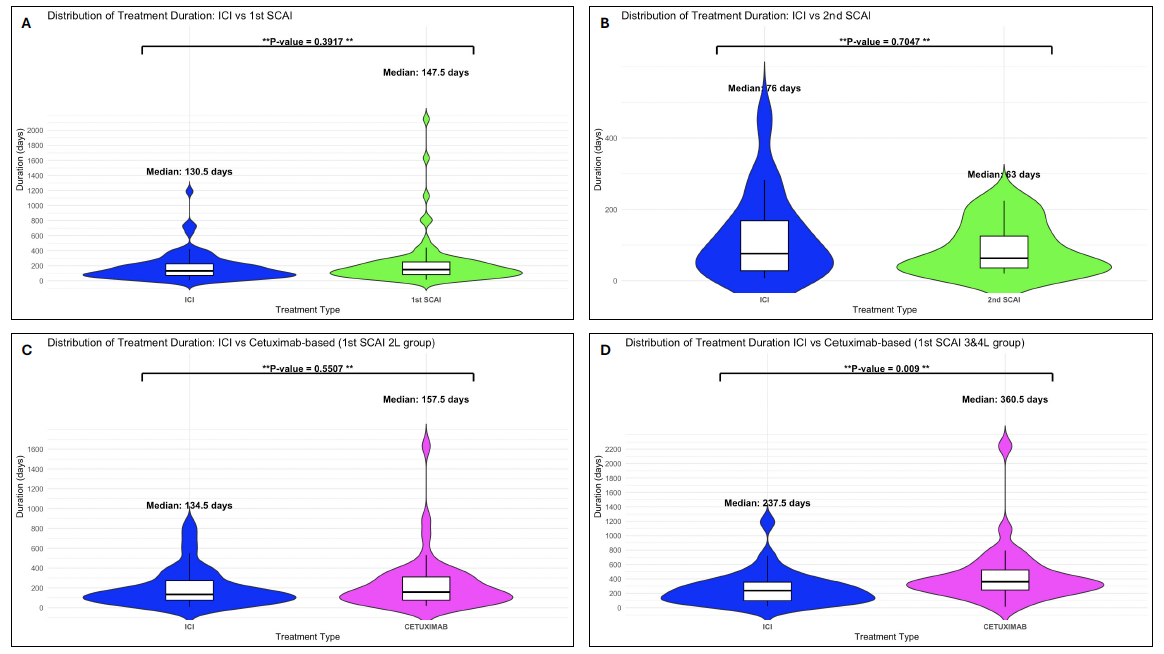
**

**Figure S12.** Violin plots depicting the comparison of treatment duration between ICI pre-SCAI and SCAI. Comparison of treatment duration between ICI pre-1^st^ SCAI and 1^st^ SCAI (A) and between ICI pre-2^nd^ SCAI and 2^nd^ SCAI (B). Comparison of treatment exposure to ICI and Cetuximab since 1^st^ line of therapy in patients treated with 1^st^ SCAI as a 2^nd^ line (C) and as 3^rd^ or 4^th^ lines (D).

**TABLES**

**Table S1.** Univariate and multivariate analyses of progression-free survival according to demographic and clinical factors.

| **Outcome** | **Variable** | **HR_Uni** | **CI_Uni** | **p-value_Uni** | **HR_Multi** | **CI_Multi** | **p-value_Multi** |
| --- | --- | --- | --- | --- | --- | --- | --- |
| **PFS 1st SCAI** | 1st SCAI (Erbitax vs Other) | 3.11 | (1.19-8.14) | 21 | 1.22 | (0.07-21.59) | 894 |
|  | 1st SCAI (weekly vs three-weekly) | 2.76 | (0.34-22.12) | 339 | 12.44 | (0.02-10161.62) | 461 |
|  | 1st SCAI Line (2nd vs 3rd-4th) | 0.37 | (0.12-1.13) | 82 | 0.53 | (0.05-5.35) | 591 |
|  | Alcohol (Yes vs No) | 3 | (1.03-8.73) | 43 | 12.78 | (0.21-781.14) | 225 |
|  | Anti-PD(L)1 (Anti-PD1 vs Anti-PDL1) | 0.46 | (0.06-3.51) | 454 | 0.86 | (0.02-39.83) | 939 |
|  | CRT (Yes vs No) | 0.62 | (0.2-1.9) | 0.4 | 0.62 | (0.03-13.06) | 756 |
|  | CRT type (Cisplatin vs Cetuximab) | 0.61 | (0.23-1.61) | 315 | 1.12 | (0.05-26.16) | 942 |
|  | ECOG PS (1 vs 2) | 0.74 | (0.28-1.95) | 539 | 0.36 | (0.04-3.59) | 382 |
|  | HPV OPC (Positive vs Negative) | 1.78 | (0.4-7.95) | 452 | 8.18 | (0.37-179.6) | 182 |
|  | Induction CT (Yes vs No) | 1.18 | (0.41-3.42) | 759 | 2.42 | (0.08-69.39) | 605 |
|  | Overall ORR (<50% vs >=50%) | 1.79 | (0.61-5.19) | 287 | 0.49 | (0.02-10.36) | 647 |
|  | Overall ORR (Response vs No Response) | 0.51 | (0.11-2.27) | 375 | 1.96 | (0.04-93.17) | 733 |
|  | PDL1 CPS (<20 vs >=20) | 0.4 | (0.15-1.04) | 59 | 0.03 | (0-0.49) | 15 |
|  | Pre-SCAI Line (ICI vs ICI + CT) | 4.07 | (1.54-10.71) | 5 | 2.15 | (0.52-8.94) | 292 |
|  | Primary Tumor (Oral cavity vs Other) | 1.3 | (0.48-3.54) | 606 | 0.28 | (0.02-3.47) | 325 |
|  | RT alone (Yes vs No) | 5.74 | (0.74-44.22) | 94 | 58.87 | (0.14-24461.88) | 185 |
|  | Relapse (Platinum-Refractory vs Platinum-Sensitive) | 1.25 | (0.35-4.41) | 728 | 4.35 | (0.3-62.84) | 0.28 |
|  | Sex (Male vs Female) | 2.18 | (0.83-5.7) | 113 | 4.55 | (0.23-90.23) | 0.32 |
|  | Smoking (Yes vs No) | 2.38 | (0.85-6.66) | 99 | 3.63 | (0.22-59.15) | 366 |
|  | Stage (Localized (I-II) vs Locally advanced (III-IVA-B)) | 0.39 | (0.08-1.83) | 235 | 0.04 | (0-2.89) | 141 |
|  | Surgery (Yes vs No) | 1.19 | (0.42-3.39) | 744 | 2.96 | (0.1-87.74) | 531 |

CI: confidence interval, CRT: chemoradiotherapy, CT: chemotherapy, CPS: combined positive score, ECOG PS: Eastern Cooperative Oncology Group Performance Status, HPV: human papillomavirus, HR: hazard ratio, ICI: immune checkpoint inhibitors, Multi: multivariate analysis, OPC: oropharyngeal cancer, ORR: objective response rate, OS: overall survival, PFS: progression-free survival, RT: radiotherapy, SCAI: salvage chemotherapy after immunotherapy, Uni: univariate analysis.

**Table S2.** Univariate and multivariate analyses of overall survival since 1^st^ SCAI according to demographic and clinical factors.

| **Outcome** | **Variable** | **HR_Uni** | **CI_Uni** | **p-value_Uni** | **HR_Multi** | **CI_Multi** | **p-value_Multi** |
| --- | --- | --- | --- | --- | --- | --- | --- |
| **OS 1st SCAI** | 1st SCAI (Erbitax vs Other) | 2.72 | (1-7.35) | 49 | 0.65 | (0.04-9.6) | 751 |
|  | 1st SCAI (weekly vs three-weekly) | 0.65 | (0.08-4.94) | 675 | 0.14 | (0-11.84) | 388 |
|  | 1st SCAI Line (2nd vs 3rd-4th) | 0.61 | (0.22-1.72) | 0.35 | 0.35 | (0.06-2.07) | 246 |
|  | Alcohol (Yes vs No) | 1.68 | (0.65-4.37) | 286 | 2.31 | (0.08-62.97) | 619 |
|  | Anti-PD(L)1 (Anti-PD1 vs Anti-PDL1) | 0 | (0-Inf) | 998 | 0 | (0-Inf) | 998 |
|  | CRT (Yes vs No) | 0.69 | (0.24-1.94) | 482 | 2.27 | (0.12-43.37) | 587 |
|  | CRT type (Cisplatin vs Cetuximab) | 1.41 | (0.52-3.82) | 494 | 3.73 | (0.19-74.02) | 387 |
|  | ECOG PS (1 vs 2) | 0.58 | (0.21-1.63) | 303 | 0.21 | (0.02-2.7) | 233 |
|  | HPV OPC (Positive vs Negative) | 1.05 | (0.24-4.63) | 0.95 | 1.12 | (0.08-15.32) | 931 |
|  | Induction CT (Yes vs No) | 0.84 | (0.29-2.44) | 744 | 2.16 | (0.11-41.42) | 609 |
|  | Overall ORR (<50% vs >=50%) | 1.54 | (0.52-4.58) | 0.44 | 0.08 | (0-2.22) | 137 |
|  | Overall ORR (Response vs No Response) | 0.42 | (0.09-1.91) | 264 | 0.34 | (0.02-6.75) | 481 |
|  | PDL1 CPS (<20 vs >=20) | 0.79 | (0.31-2.01) | 618 | 0.11 | (0.01-1.08) | 58 |
|  | Pre-SCAI Line (ICI vs ICI + CT) | 4.42 | (1.64-11.89) | 3 | 2.18 | (0.52-9.09) | 284 |
|  | Primary Tumor (Oral cavity vs Other) | 1.33 | (0.49-3.59) | 576 | 0.5 | (0.04-5.87) | 579 |
|  | RT alone (Yes vs No) | 2.91 | (0.66-12.92) | 159 | 20.45 | (0.61-683.48) | 92 |
|  | Relapse (Platinum-Refractory vs Platinum-Sensitive) | 2.87 | (0.64-12.88) | 169 | 5.12 | (0.31-84.19) | 253 |
|  | Sex (Male vs Female) | 2.12 | (0.78-5.75) | 139 | 29.24 | (1.76-485.39) | 19 |
|  | Smoking (Yes vs No) | 1.51 | (0.58-3.96) | 401 | 0.67 | (0.03-13.69) | 793 |
|  | Stage (Localized (I-II) vs Locally advanced (III-IVA-B)) | 0.51 | (0.11-2.35) | 0.39 | 0.09 | (0-2.35) | 0.15 |
|  | Surgery (Yes vs No) | 1.38 | (0.49-3.89) | 543 | 2.35 | (0.15-36.93) | 544 |

CI: confidence interval, CRT: chemoradiotherapy, CT: chemotherapy, CPS: combined positive score, ECOG PS: Eastern Cooperative Oncology Group Performance Status, HPV: human papillomavirus, HR: hazard ratio, ICI: immune checkpoint inhibitors, Multi: multivariate analysis, OPC: oropharyngeal cancer, ORR: objective response rate, OS: overall survival, RT: radiotherapy, SCAI: salvage chemotherapy after immunotherapy, Uni: univariate analysis.

**Table S3.** Univariate and multivariate analyses of overall survival since 1^st^ line according to demographic and clinical factors.

| **Outcome** | **Variable** | **HR_Uni** | **CI_Uni** | **p-value_Uni** | **HR_Multi** | **CI_Multi** | **p-value_Multi** |
| --- | --- | --- | --- | --- | --- | --- | --- |
| **OS 1st Line** | 1st SCAI (Erbitax vs Other) | 3.06 | (1.07-8.81) | 38 | 2.01 | (0.26-15.58) | 503 |
|  | 1st SCAI (weekly vs three-weekly) | 0.78 | (0.1-6) | 0.81 | 0.29 | (0-17.04) | 0.55 |
|  | 1st SCAI Line (2nd vs 3rd-4th) | 0.33 | (0.12-0.92) | 34 | 0.12 | (0.02-0.63) | 12 |
|  | Alcohol (Yes vs No) | 1.74 | (0.67-4.51) | 254 | 1.3 | (0.06-30.34) | 869 |
|  | Anti-PD(L)1 (Anti-PD1 vs Anti-PDL1) | 0 | (0-Inf) | 998 | 0 | (0-Inf) | 998 |
|  | CRT (Yes vs No) | 0.94 | (0.32-2.72) | 908 | 5.11 | (0.36-72.05) | 227 |
|  | CRT type (Cisplatin vs Cetuximab) | 1.21 | (0.45-3.27) | 707 | 2.44 | (0.15-39.97) | 531 |
|  | ECOG PS (1 vs 2) | 0.79 | (0.3-2.07) | 628 | 1.39 | (0.21-9.37) | 732 |
|  | HPV OPC (Positive vs Negative) | 0.62 | (0.14-2.75) | 534 | 0.9 | (0.08-10.67) | 931 |
|  | Induction CT (Yes vs No) | 1.02 | (0.35-2.99) | 973 | 0.64 | (0.03-13.11) | 0.77 |
|  | Overall ORR (<50% vs >=50%) | 1.66 | (0.55-5) | 368 | 0.54 | (0.03-8.88) | 663 |
|  | Overall ORR (Response vs No Response) | 0.32 | (0.07-1.46) | 142 | 0.71 | (0.03-17.41) | 833 |
|  | PDL1 CPS (<20 vs >=20) | 0.96 | (0.37-2.46) | 927 | 0.55 | (0.08-3.67) | 535 |
|  | Pre-SCAI Line (ICI vs ICI + CT) | 6.49 | (2.22-18.98) | 1 | 6.52 | (1.26-33.64) | 25 |
|  | Primary Tumor (Oral cavity vs Other) | 0.86 | (0.33-2.26) | 765 | 0.7 | (0.07-6.63) | 0.76 |
|  | RT alone (Yes vs No) | 2.17 | (0.49-9.72) | 0.31 | 1.72 | (0.06-47.29) | 748 |
|  | Relapse (Platinum-Refractory vs Platinum-Sensitive) | 1.93 | (0.44-8.49) | 383 | 1.57 | (0.1-24.15) | 748 |
|  | Sex (Male vs Female) | 1.36 | (0.53-3.49) | 528 | 2.23 | (0.3-16.46) | 0.43 |
|  | Smoking (Yes vs No) | 1.72 | (0.66-4.46) | 263 | 0.66 | (0.03-14.17) | 794 |
|  | Stage (Localized (I-II) vs Locally advanced (III-IVA-B)) | 0.42 | (0.09-2.03) | 281 | 0.14 | (0.01-2.09) | 154 |
|  | Surgery (Yes vs No) | 1.19 | (0.42-3.41) | 745 | 2.08 | (0.15-29.41) | 589 |

CI: confidence interval, CRT: chemoradiotherapy, CT: chemotherapy, CPS: combined positive score, ECOG PS: Eastern Cooperative Oncology Group Performance Status, HPV: human papillomavirus, HR: hazard ratio, ICI: immune checkpoint inhibitors, Multi: multivariate analysis, OPC: oropharyngeal cancer, ORR: objective response rate, OS: overall survival, RT: radiotherapy, Uni: univariate analysis.

**Table S4.** Safety summary during 1^st^ and 2^nd^ SCAI

| **1^st^ SCAI** | **Grade 1** | **Grade 2** | **Grade 3** | **Grade 4** | **Grade 5** |
| --- | --- | --- | --- | --- | --- |
| Rash | 5 (6.3%) | 46 (57.5%) | 5 (6.3%) | 1 (1.3%) | 0 |
| Pruritus | 1 (1.3%) | 3 (3.8%) | 1 (1.3%) | 0 | 0 |
| Mucositis | 4 (5%) | 29 (36.3%) | 2 (2.5%) | 0 | 0 |
| Skin fissures | 2 (2.5%) | 7 (8.8%) | 0 | 0 | 0 |
| Paronychia | 3 (3.8%) | 1 (1.3%) | 0 | 0 | 0 |
| Conjunctivitis | 5 (6.3%) | 7 (8.8%) | 0 | 0 | 0 |
| Ectropion | 1 (1.3%) | 2 (2.5%) | 0 | 0 | 0 |
| HypoMg++ | 6 (7.5%) | 30 (37.5%) | 0 | 0 | 0 |
| Anorexia | 3 (3.8%) | 3 (3.8%) | 0 | 0 | 0 |
| Asthenia | 6 (7.5%) | 21 (26.3%) | 0 | 0 | 0 |
| Diarrhea | 3 (3.8%) | 4 (5%) | 1 (1.3%) | 2 (2.5%) | 0 |
| Alopecia | 4 (5%) | 28 (35%) | 0 | 0 | 0 |
| Peripheral neuropathy | 2 (2.5%) | 12 (15%) | 1 (1.3%) | 0 | 0 |
| Anemia | 6 (7.5%) | 31(38.8%) | 1 (1.3%) | 0 | 0 |
| Neutropenia | 3 (3.8%) | 23 (28.8%) | 10 (12.5%) | 2 (2.5%) | 0 |
| Thrombopenia | 0 | 11 (13.8%) | 2 (2.5%) | 1 (1.3%) | 0 |
| URTI | 0 | 29 (36.3%) | 0 | 0 | 0 |
| LRTI | 0 | 3 (1.3%) | 4 (8.8%) | 0 | 0 |
| PEG infection | 0 | 1 (1.3%) | 0 | 0 | 0 |
| Sepsis | 0 | 1 (1.3%) | 0 | 0 | 1 |
| Nausea | 1 (1.3%) | 7 (8.8%) | 0 | 0 | 0 |
| Vomiting | 3 (3.8%) | 2 (2.5%) | 0 | 0 | 0 |
| Renal insufficiency | 0 | 1 (1.3%) | 0 | 0 | 0 |
| Tumor bleeding | 0 | 5 (6.3%) | 5 (6.3%) | 0 | 0 |
| Peptic ulcer bleeding | 0 | 0 | 1 (1.3%) | 0 | 0 |
| Total incidence | G1-2: 80/80 (100%) | | G3-5: 31/80 (38.7%) | | |
| **2^nd^ SCAI** | **Grade 1** | **Grade 2** | **Grade 3** | **Grade 4** | **Grade 5** |
| Rash | 4 (23.5%) | 8 (47.1%) | 1 (5.9%) | 0 | 0 |
| Pruritus | 0 | 1 (5.9%) | 0 | 0 | 0 |
| Mucositis | 2 (11.8%) | 5 (29.4%) | 1 (5.9%) | 0 | 0 |
| Skin fissures | 0 | 1 (5.9%) | 0 | 0 | 0 |
| Paronychia | 0 | 1 (5.9%) | 0 | 0 | 0 |
| Conjunctivitis | 1 (5.9%) | 0 | 0 | 0 | 0 |
| HypoMg++ | 0 | 3 (17.6%) | 0 | 0 | 0 |
| Anorexia | 0 | 2 (11.8%) | 0 | 0 | 0 |
| Asthenia | 3 (17.6%) | 3 (17.6%) | 0 | 0 | 0 |
| Diarrhea | 0 | 0 | 0 | 0 | 0 |
| Alopecia | 1 (5.9%) | 3 (17.6%) | 0 | 0 | 0 |
| Peripheral neuropathy | 1 (5.9%) | 2 (11.8%) | 0 | 0 | 0 |
| Anemia | 2 (11.8%) | 10 (58.8%) | 2 (11.8%) | 0 | 0 |
| Neutropenia | 0 | 1 (5.9%) | 1 (5.9%) | 1 (5.9%) | 0 |
| Thrombopenia | 0 | 1 (5.9%) | 0 | 1 (5.9%) | 0 |
| URTI | 0 | 4 (23.5%) | 0 | 0 | 0 |
| LRTI | 0 | 1 (5.9%) | 5 (29.4%) | 0 | 0 |
| Sepsis | 0 | 0 | 2 (11.8%) | 0 | 0 |
| Bacteriemia | 0 | 0 | 1 (5.9%) | 0 | 0 |
| Nausea | 0 | 7 (41.2%) | 1 (5.9%) | 0 | 0 |
| Vomiting | 0 | 3 (17.6%) | 0 | 0 | 0 |
| Tumor bleeding | 0 | 2 (11.8%) | 1 (5.9%) | 0 | 0 |
| Total incidence | G1-2: 17/17 (100%) | | G3-5: 8/17 (47%) | | |

G1-2: grade 1-2, G3-5: grade 3-5, HypoMg++: hypomagnesemia, LRTI: lower respiratory tract infection, SCAI: salvage chemotherapy after immunotherapy, URTI: upper respiratory tract infection
